# Progress on the Fabrication and Application of Electrospun Nanofiber Composites

**DOI:** 10.3390/membranes10090204

**Published:** 2020-08-28

**Authors:** Mariela Toriello, Morteza Afsari, Ho Kyong Shon, Leonard D. Tijing

**Affiliations:** 1 Faculty of Engineering and Information Technology, University of Technology Sydney (UTS), 15 Broadway, Ultimo, NSW 2007, Australia; marielatm9@gmail.com; 2 Centre for Technology in Water and Wastewater, School of Civil and Environmental Engineering, University of Technology Sydney (UTS), 15 Broadway, Ultimo, NSW 2007, Australia; Morteza.Afsari@student.uts.edu.au (M.A.); hokyong.shon-1@uts.edu.au (H.K.S.)

**Keywords:** electrospinning, nanofiber, composites, membrane, environment, health, energy

## Abstract

Nanofibers are one of the most attractive materials in various applications due to their unique properties and promising characteristics for the next generation of materials in the fields of energy, environment, and health. Among the many fabrication methods, electrospinning is one of the most efficient technologies which has brought about remarkable progress in the fabrication of nanofibers with high surface area, high aspect ratio, and porosity features. However, neat nanofibers generally have low mechanical strength, thermal instability, and limited functionalities. Therefore, composite and modified structures of electrospun nanofibers have been developed to improve the advantages of nanofibers and overcome their drawbacks. The combination of electrospinning technology and high-quality nanomaterials via materials science advances as well as new modification techniques have led to the fabrication of composite and modified nanofibers with desired properties for different applications. In this review, we present the recent progress on the fabrication and applications of electrospun nanofiber composites to sketch a progress line for advancements in various categories. Firstly, the different methods for fabrication of composite and modified nanofibers have been investigated. Then, the current innovations of composite nanofibers in environmental, healthcare, and energy fields have been described, and the improvements in each field are explained in detail. The continued growth of composite and modified nanofiber technology reveals its versatile properties that offer alternatives for many of current industrial and domestic issues and applications.

## 1. Introduction

Electrospun nanofiber technology has gained tremendous interest due to its versatility and the possibility to fabricate many designs and functionalities from various materials and strategies. In the technical definition, nanofibers are fibers with diameters in the range of tens of nanometers to less than 1 µm. In other words, they have a very high aspect ratio and are observed as one-dimensional materials. These features give them unique properties which can be applied in various applications. They also have unlimited assembly configurations and functionalities depending on the solution properties, environmental factors, and technical variables [1]. Nanofibers are produced in various ways such as wet-spinning [2], dry-spinning or melt spinning [3,4], template synthesis [5], solution blow spinning [6], and force spinning [7], but the most common and highly effective way is through an electrospinning (electrostatic spinning) technique, i.e., the fabrication of nanofibers using electrostatic forces [8]. Electrospun nanoscale fibers are mainly fabricated from polymer solution or polymer melts [9]. Though electrospinning has advanced rapidly in the past three decades, the technique itself and the fabrication parameters still need to be enhanced and improved to match commercial requirements. For example, obtaining clog-free electrospun nanofibers is still a challenge [9]. However, with technological advancements and extensive research, electrospinning technology has evolved from lab-made nanofibers to more advanced products. Extensive research has led to the fabrication of nanofibers with increased strength, more controllable morphology (length, orientation, porosity, diameter, shape, size, and alignment), and new functionalities [9]. One aspect for improvement would be the physicochemical characteristics of the nanofibers by appropriately selecting basic materials to enhance porosity and durability. Nanofibers also generally have low mechanical strength because of their low crystallinity and random alignment and orientation [10]. The layering and thickness increment leads to formation of a nonwoven or membrane structure and enhances the functionalities of the nanofibers [10]. Nanofiber membranes are popular where electrical, mechanical, and thermal properties as well as separation processes are required, due to their capacity to play the role as a host to immobilize nanoparticles, as adsorbent material for desired pollutants, in mimicking the extracellular matrix for tissue regeneration, and as electrode for batteries and energy devices [9,11]. They also improve both separation and mechanical characteristics for applications like water purification, desalination [12], and removal of viruses, bacteria, and toxic metal ions from water. This is why many research groups are developing electrospun nanocomposites that can benefit from the properties of more than one material and, at the same time, can have the advantages granted by nanoscale features [1].

Electrospun nanofiber-based composites are a growing type of materials that can provide different and diverse properties and functionalities. They have the advantage of polymers in being flexible, easy to fabricate, cheap, easy to mold, and lightweight [13,14]. In addition, the combination of various materials in the polymer matrix adds functionalities, giving the desirable mechanical and chemical characteristics of inorganic materials like high thermal stability and strength [15,16], and use of special polymers and metal nanoparticles can also render conductivity to the composite [17]. The selection of materials is highly important to achieve the desired functionality and complexity for various applications [10]. However, pristine nanofibers are limited in their functionalities and, thus, different techniques to modify the chemical composition and surface morphology of electrospun nanofibers (ENFs) have been developed. These include coating, decorating, and functionalizing techniques using materials like metals, nanoparticles, biomolecules, enzymes, carbon nanotubes, surfactants, and polymers [18]. The post-treatment modifications can include functionalization of the nanofiber membrane outer surface, modification of the nanofiber structure, hot-press treatment [19], or development of thin-film nanofiber composite (TFNC) membranes [16].

**Figure 1:**
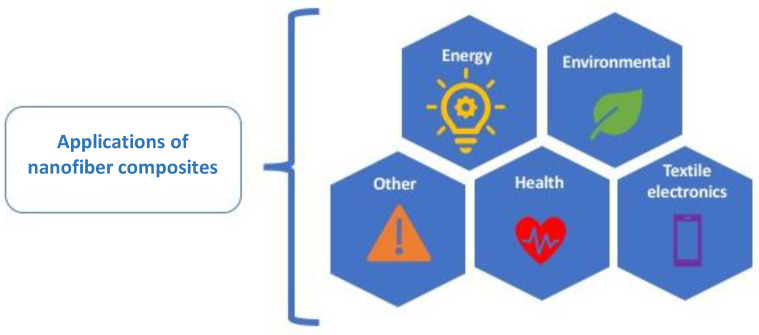
Various applications of electrospun nanofiber composites: environmental, health, energy, textile electronics, and other.

To understand the limitations of electrospun composite and modified nanofibers and the development they have gone through, the process of their preparation and fabrication are addressed in this review. A quick check in the literature shows a sharp increase in studies about electrospun composites and modified nanofibers, especially in the past decade. The potential of electrospun nanofiber composites is enormous, but more studies are needed to integrate both robust performance and economy. This literature review describes the different applications of nanofiber composites with emphasis on new application developments, knowing there is still a lot to be explored and improved. The advantages and disadvantages of electrospun composite nanofibers in various applications are discussed here in detail. Unlike other reviews, this review is more focused on composite and modified nanofiber materials (as opposed to pristine nanofibers) from their fabrication, material types, nanofiber structures, and multifunctionalities toward a wide range of applications, including environmental, health, energy, textile electronics, and others (see Figure 1). It is hoped that this review will provide new insights on nanofiber composites and modification and provide ideas for future research directions.

## 2. Overview of Electrospinning and Electrospun Nanofiber Composites

Electrospinning is a facile technique to fabricate ultrafine fibers with long length and narrow diameter. The electrospinning equipment consists of a syringe or capillary tube serving as a container for polymer solution, a metallic needle (or spinneret) for jetting of solution, a high-voltage power supply (10–60 kV), and a grounded collector for collecting nanofibers [20]. A high-voltage power supply induces an electrical field between the polymer solution in a spinneret and the collector (e.g., rotating drum or a flat plate) separated by a suitable distance [21]. A peristaltic pump drives the solution from the needle and ejects the droplet at the tip of the needle, which is connected to a high-voltage power supply that induces charge to the droplet [22,23]. The surface tension of the polymer solution is overcome by the electrical force, elongating the droplet and forming a cone, resulting in the ejection of a thin jet that stretches and further elongates due to the combination of charge repulsion and solvent evaporation [1,20,24]. This elongated thin jet then solidifies into fibers, directly depositing onto a counter electrode collector [21,22]. Conventionally, nanofibers are in the form of pristine fibers, where only one component polymer is used in the process. To add functionalities and to enhance the application of nanofibers, electrospun nanofiber composites and their modification are prepared and are usually approached in the following ways:

(1) Fabrication of mixed-matrix composite nanofibers (a solution containing polymer and dispersed inorganic fillers (e.g., ZnO, TiO_2_, carbon nanotubes, graphene oxide, etc.);
(2) Production of nanofibers utilizing two or more precursors and fabrication of core–shell nanofiber or bi- or multicomponent-based composite nanofibers, and;
(3) Fabrication of polymeric nanofiber and then subsequent post-treatment of the surface to produce composite electrospun nanofiber.

The desired design and morphology of the resulting nanofiber composites can be controlled by manipulating electrospinning parameters (materials and processes) and also by the post-treatment modification process [20]. Material parameters include properties of the polymer solution (molecular weight, viscosity, electrical conductivity, conformation of polymer chains, surface tension, and solvent type) while the process parameters include applied voltage, distance between the needle and collector, electrode geometry, rotating speed of the collector, feeding rate of the polymer solution, and the environmental conditions (temperature and humidity) [21]. The post-treatment modification includes dip-coating, hot-press treatment, layer-by-layer surface modification, plasma treatment, etc.

**Figure 2:**
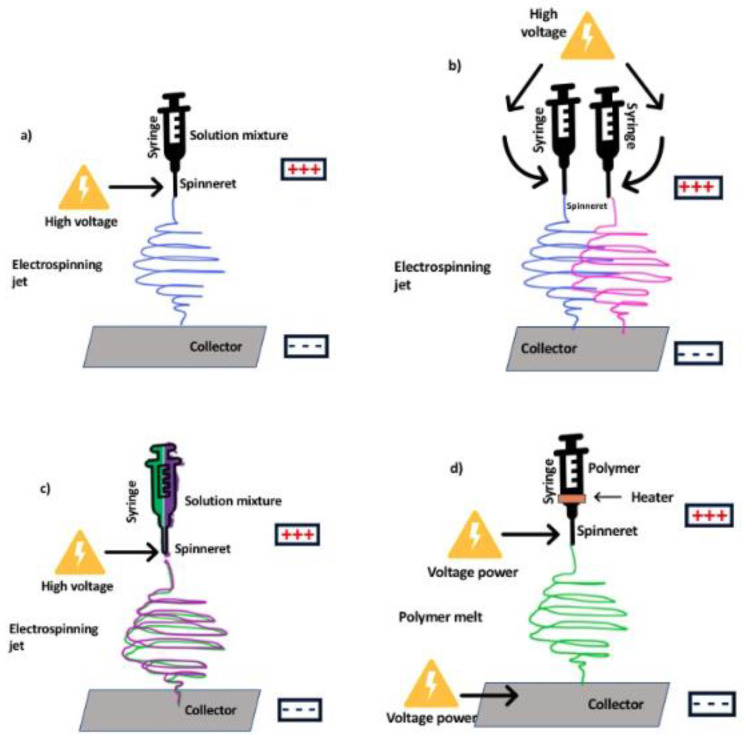
Various kinds of electrospinning setups for nanofiber fabrication: (**a**) conventional one-nozzle solution electrospinning; (**b**) dual or multi-nozzle; (**c**) Side-by-side electrospinning, and; (**d**) melt electrospinning.

There are many types of electrospinning configurations based on the type and design of the spinneret and collector (see Figure 2). The spinneret can be a single or multi-spinneret or have a coaxial or triaxial design. Other configurations use a needleless setup [25]. Variations can also be in the number of nozzles and based on the nature and number of axial units (coaxial, monoaxial, and multiaxial). Single-nozzle equipment is used for easily soluble solutions and multi-nozzles have the advantage of large fiber production and multicomponent production [22,26]. In the case of different types of collectors (e.g., parallel plate, drum collector, plate collector, cocoon and disc collector), the choice depends on the application associated with the fabricated nanofiber [22]. For example, a disc collector with high rotational speed can be used for the fabrication of aligned nanofibers.

**Figure 3:**
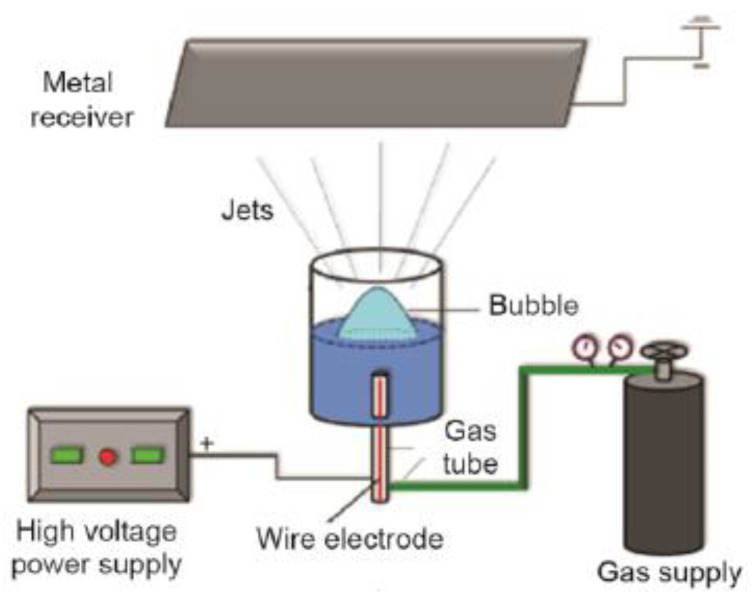
Schematic illustration of a critical bubble electrospinning system [27].

Another electrospinning system which is designed for potential mass production is critical bubble electrospinning. In this method, electrospinning starts from multiple points on the surface of a single bubble, and nanofiber threads are fabricated simultaneously. As shown in Figure 3, a gas pressure creates bubbles on the surface of a solution container, which is connected to a high-voltage supply. Raising the surface of the solution in the bubble causes the nanofibers to commence jetting toward the collector surface. Accurate voltage and gas pressure are crucial parameters to create a bubble in its critical point, i.e., the point where the bubble is in its bursting state. At this point, the solution thickness on the bubble surface is at its minimum value and the surface tension can be overcome by applied electrical force to eject jets [27].

Electrospinning has the advantages of providing a high level of fabrication flexibility to control the nanofiber microstructure, arrangement, and diameter, and also benefits from the option of wide material selection, easy incorporation of additives, straightforward process, and a practical way to generate nanostructures [16]. However, electrospinning has deficiency for providing functional groups on the surface for some special applications and, therefore, post-electrospinning modification (chemical or physical post-treatment) is essential to decorate the membrane surface with desired nanoparticles to increase its active sites and functionality. Physical modifications usually have poor performance and, therefore, chemical methods like grafting, hydrolysis, oxidation, aminolysis, and crosslinking are being used [18]. Other strategies for modifying electrospun membranes using nanotechnology are layer-by-layer deposition, molecular imprinting, sol–gel technique, and atomic deposition, which provide functional coating of different materials on the surface of nanofiber membranes. In many cases, a composite structure wherein nanofibers are imparted with additional properties are used so as to expand its properties and potential use. 3D nanofiber structures have also garnered increasing research due to their potential applicability in tissue engineering and even for filtration [28]. The 3D structure can be fabricated by sequential electrospinning for a long time to increase the thickness of the scaffold. Another way is via a gas-foaming technique, wherein the fabricated nanofibers are exposed to special gases that make them swell and expand to form a low-density, sponge-like structure. Bioactive inorganic nanoparticles can be integrated to form nanofiber composites and provide additional functionalities, which are especially interesting for biomedical applications. The next section gives details of the various approaches of producing nanofiber composites.

## 3. Types of Electrospun Nanofiber Composites

### 3.1. Electrospun Mixed-Matrix Nanofibers and Nanocomposite Membranes

**Figure 4:**
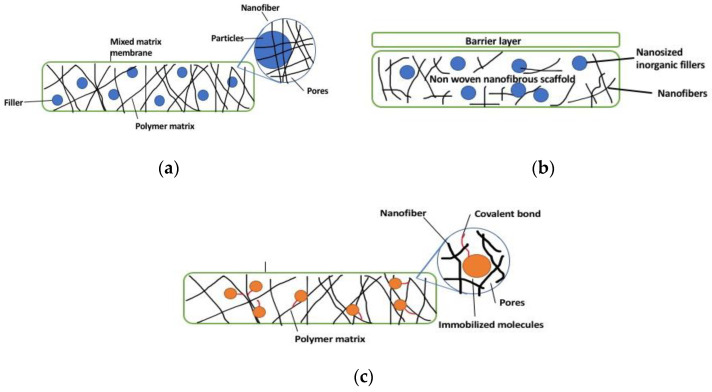
(**a**) Mixed-matrix nanocomposite, (**b**) hybrid nanocomposite, and (**c**) surface-functionalized nanocomposite membranes.

Mixed-matrix nanofibers use inorganic materials mixed into a polymer solution for nanofiber formation [29]. In other words, mixed-matrix nanofibers have a polymeric matrix and use inorganic particles (usually inorganic particles with high porosity) as dispersed fillers, combining the properties of inorganic fillers and organic matrix, resulting in improvement of overall properties (Figure 4a) [30,31]. The nanofiber composites are usually nonwoven and in the form of membranes which, in this case, are generally known as mixed-matrix membranes (MMMs). MMMs have the advantage of possessing the physicochemical stability of ceramics and the easy formation of polymeric materials on top of high resistance to fouling; high thermal, chemical, and mechanical strength; wider pH and temperature range; and high permselectivity [32,33]. Nanoparticles can be self-assembled within the polymer matrix via the condensation and hydrolysis reaction of inorganic precursors [34]. Physical blending of nanoparticles can also produce nanocomposite membranes, wherein it requires dissolution in a solvent or melt mixing to disperse the inorganic nanoparticles in the dissolved polymer. However, uniform dispersion is hard to obtain using this method [34]. The incorporation of nanoparticles into polymer matrices can also be done by hot-press techniques. Nonetheless, both hot-press and phase-inversion techniques have the challenge of uniform distribution and dispersion of the nanoparticles in/on the membrane [34].

### 3.2. Thin-Film Nanofiber Composite and Hybrid Membranes

Thin-film nanofiber composite (TFNC) membranes have multiple layers: a support layer, one nonwoven nanofiber middle layer, and a top layer as a barrier (Figure 4b) [35]. This design has been tested in membrane separation processes, wherein the nanofiber layer is utilized as a support structure. TFNC membranes can also incorporate a polymeric nanofiber support matrix with nano-sized inorganic fillers, such as titanium dioxide, silica, nanoclays, and zirconium dioxide, which have resulted in improved properties of the membrane for ionic conductivity and water retention [36]. Hybrid nanofiber-based membranes refer to membranes that are made up of two or more layers of either all nanofiber layers or a combination of nanofibers and casted or polymerized layers. For the hybrid fabrication process, successive electrospinning (two nozzles, coaxial, and rotational) of two or more polymers has been the common method to use due to its simplicity, speed, and cost-effectiveness [37,38]. This results in a build-up of nanofiber layers on top of each other that may have different properties, such as the production of Janus nanofiber membranes (i.e., two sides of the membrane have different wettability). However, hybrid membranes can suffer from delamination due to the incompatibility of the various layers.

### 3.3. Surface-Functionalized Nanofiber Composites

Surface functionalization offers high binding affinity, improved separation performance, and increased loading capacity to overcome the challenge of binding and compatibility of nanoparticle with the polymer matrix [39]. Surface-functionalized nanofibers have the advantage of having covalently attached immobilized molecules, therefore avoiding them to be easily separated from the surface over an extended period of time (Figure 4c). Nonetheless, partial inactivation can occur to the immobilized molecules in the situation that active sites are being chemically modified [40]. Methods to modify the surface of nanofibers with cell recognizable ligands and bioactive molecules include wet chemical method, surface graft polymerization, hot-pressing, plasma treatment, and co-electrospinning [40]. These methods chemically and physically modify the surface of membranes to obtain superb functionalities [40].

Plasma treatment modifies the chemical composition of the surface to tailor wetting properties and surface adhesion of the membrane without affecting the bulk characteristics [18]. The appropriate selection of plasma allows the introduction of functional groups to improve biocompatibility or to allow various bioactive molecules to be covalently immobilized on the target surface [40]. However, plasma treatment cannot effectively modify the surface of nanofibers that are located at the core of the membrane due to the penetration limit of the plasma [40]. After plasma treatment, it is possible to add chemically reactive functional groups; however, this often results in difficulty in acquiring surface functionalization with just one group of functional groups. In addition, it generates several reactive species on the surface, limiting its specificity [18]. Through partial hydrolysis of polyester films under basic or acidic conditions, one study showed that a wet chemical method can offer surface modification to modify surface wettability or add new functionality for thick nanofiber membranes. In partial hydrolysis, the duration of the process and the concentration of the hydrolyzing agent determine the effectiveness of the functional groups on the surface without drastically changing the bulk properties [40,41]. Tiraferri et al. demonstrated that the increase in hydrogen bonding sites in the membrane surface results in maximized interfacial acid–base forces. Therefore, water molecules having tightly bonded interfacial layers with high orientation and slow dynamics are formed [42]. Since most biodegradable polymers retain their hydrophobicity, surface graft polymerization can be used to transfer hydrophilicity to the surface and for the introduction of multifunctional groups by covalent immobilization of bioactive molecules [43]. Surface grafting is the process where a polymer or other chemical chain grows by chemical linkage on electrospun nanofiber substrate [44]. It commences with UV radiation and plasma treatment, leading to the creation of free radicals which are used in the polymerization process [40].

Another method for improving hydrophilicity is by chemical crosslinking, which stabilizes the nanofibers with the promotion of coupling and bonding reactions between polymer chains [45]. Electrospun nanofibers can also be modified by surface coating or physical vapor deposition, which refers to an electrospun membrane surface being coated with a thin inorganic film to increase surface conductivity. This modification method includes heating, evaporation, and deposition. However, since the method is limited to inorganic coating, the attachment of active groups is not durable [46]. Thermal treatment is another way to modify the nanofiber membranes and can enhance the mechanical properties and structural integrity via the formation of fused fibrous structures. However, this treatment can cause damage on the fiber after prolonged treatment [18,46]. A co-electrospinning technique is an attractive way to functionalize the surface by a one-step electrospinning process. With the use of a coaxial nozzle, a solution containing functional groups is placed at the sheath layer of the coaxial nozzle, thus directly exposing the functional polymer segments and nanoparticles to the surface of the nanofibers during fabrication [47].

### 3.4. Electrospun Ceramic Nanofiber Composites

Ceramics are composed of different metallic materials, although many non-metallic and biomaterial compounds can be treated to become ceramic materials. Ceramics are usually composed of oxides, sulfides, carbides, or nitrides, and most of them have a crystalline structure. Ceramics are formed from anions and cations with high attraction forces that give outstanding physical and chemical properties [23]. They are widely used in different applications (surgery and dental implants, catalysts, supports, sensors, etc.) and the fabrication of nano-based ceramic material is in high demand for the production of highly efficient materials [48]. Electrospun composite ceramic nanofibers (ECCNFs) are a special type of ENF that have the characteristics of ceramic materials, including high mechanical, thermal, and chemical resistance and catalytic and photocatalytic activity, but in the form of nanofibers (i.e., with high aspect ratio, high surface area, high porosity, low density, controllable fiber parameters, and being cost-effective). ECCNFs can be used in various industrial applications like air filtration (especially in high-temperature or corrosive conditions), water treatment, catalytic and photocatalytic activities, and so on [49].

Ceramic nanofibers are usually fabricated by the sol–gel technique and using polymer reagent routes. Polymeric solutions containing precursors are electrospun and, afterwards, the nanofibers go through chemical conversion or thermal treatment for synthesis of ceramic nanofibers. Ceramic nanofibers can be improved with hydrothermal, carbothermal, and pyrolysis post-treatment [23], wherein the post-treatment procedures can affect the structure and dimensions of the ceramic nanofibers [24,45]. Calcination at high temperature under air oxidizes the polymer functional groups, and, step by step, removes all parts of the polymer [50]. The presence of the oxygen in air then proceeds to the conversion of ceramic particles to oxides. Pyrolyzing in an inert atmosphere (N_2_ or Ar) changes the polymer structure to a carbon and graphite structure, which increases the adsorption ability of the nanofibers [51]. In this state, ceramic particles react with N_2_ and increase the nitrides in the nanofiber structure. Calcination with a high heating rate breaks the nanofibers and, thus, production of continuous ceramic nanofibers is not possible [20,52]. In one study, niobium–lithium–silica–PVP nanofibers with an average diameter of 760 nm were fabricated using a coaxial electrospinning method. After calcination, the nanofiber sizes reduced to almost half the original diameter, which is attributed to the removal of volatile parts of the nanofibers, and remaining parts reacted with each other, shrink, and form new tighter and denser structures [53].

The concentration of the ceramic precursors and polymer should be adequately controlled to produce continuous ceramic nanofiber with a desirable diameter. A high ceramic precursor concentration increases the diameter of the nanofibers and a low concentration of ceramic causes breaking of the nanofibers in the calcination step [23,54]. A new method has been introduced to incorporate ceramic or metallic nanoparticles in CNFs. In this method, after electrospinning of the solution, nanoparticles are electrosprayed onto the surface of the nanofibers. Then, through the calcination process, the nanoparticles are stabilized, and homogenous surface-treated electrospun ceramic nanofibers are fabricated. In addition, the carbonization step increases the porosity of the nanofibers and enhances the nanofiber surface area [48,51]. In one study, ZnO/In_2_O_3_ electrospun composite ceramic nanofibers were fabricated using a co-electrospinning technique [55]. The fabricated nanofibers were then calcined at different temperatures to determine the calcination temperature effect on the morphology of the nanofibers. The diameter of the nanofibers halved after the calcination process. In this study, different nozzle–collector distances were set, and results showed that a very small distance between the nozzle and collector results in poor arrangement of the nanofibers on the collector due to the low drying time for nanofibers. Investigation of the feed rate showed that it should be adjusted to an optimum value. They observed that low flow rates break the nanofibers and high flow rates eject big droplets and create beads on the nanofibers. Applied voltage impact analysis revealed that high voltage produces more homogenous nanofibers, but large defects are formed in some points of the nanofibers. Hence, the quality of the nanofibers is more greatly verified at a voltage near the lower ejection point at minimum applicable voltage [55].

In another work, electrospun ceramic nanofibers were fabricated using a non-isothermal method. In this study, colloidal silica (CS) and tetraethyl orthosilicate (TEOS) were electrospun and calcined at 1000 °C to prepare ceramic nanofibers, which, when compared to common silica nanofibers, have lower crystallinity, smaller grain size, and more grains [56]. Yang et al. [57] also prepared triaxial core–shell nanohybrids using the electrospinning process for biomedical and drug delivery application. Three solutions were used, namely the outer solvent, a middle non-electrospinnable solution (dilute cellulose acetate (CA)), and an inner (core) electrospinnable solution (ibuprofen–gliadin solution). The diameter of the triaxial system can be controlled by the adjustment of CA solution. In vitro experiments showed an initial burst release of the drug, which is controlled by the coating of CA. This coating helped the slow release of the drug for long period of time (30 days), which is suitable for moderate drug release for antibiotic activities [57].

## 4. Applications of Electrospun Nanofiber Composites

### 4.1. Environmental Applications

#### 4.1.1. Membrane Separation and Water Purification

Electrospun nanofiber composite membranes have attracted increasing interest in recent years for their application in membrane separation, desalination, water and wastewater treatment, oil/water separation, and adsorption. In separation technologies, the membrane performance is mainly based on the membrane’s flux and selectivity [58,59]. High separation performance together with properties of mechanical and chemical resistivity can affect the functionality and applicability of the membrane in industrial usage [60]. The high surface area, porous structure, and controllable pore sizes make electrospun nanofiber (ENF) membranes suitable for water-related applications [61,62]. Though pristine nanofiber membranes have shown decent performance, the nanofiber composite structures where additional properties and functionalities are provided to the membrane make them better versions. The nanofiber composites are either used as standalone, as support layer, or as hybrid materials for many membrane-based processes such as microfiltration, membrane distillation (MD), ultrafiltration (UF), nanofiltration (NF), and reverse osmosis (RO) [63].

**Figure 5:**
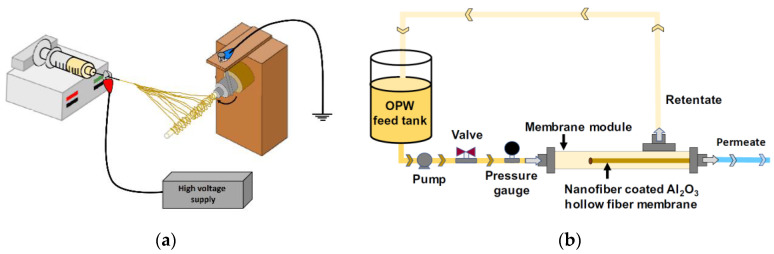
Schematic of (**a**) electrospinning setup for nanofiber-coated alumina membrane fabrication and (**b**) crossflow membrane filtration setup [67].

Nanofibers have average pore sizes between 0.1 micron to a few microns, which is the usual range for microfiltration membranes. Due to the wider pore size distribution, they cannot be used directly for brine treatment but can be used for pre-treatment, agricultural applications, removal of viruses and bacteria, etc. [64,65]. Many researchers have suggested different methods to overcome the low mechanical strength of ENFs, like using support layer, coaxial spinning, co-spinning, incorporation of nanoparticles, adjusting the solution parameters, etc. Single-nozzle or two-nozzle electrospinning technique can be used for the production of supported nanofiber composite membranes for MF application. Liu et al. [66] fabricated electrospun composite nanofibers produced from PVDF–hexafluoropropylene (HFP) copolymer coated with CuO nanoparticles for MF application. PVDF–HFP copolymer nanofibers showed superb flexibility and mechanical properties (6.7 MPa and 67% elongation at break). The coating of CuO nanoparticles was found to significantly enhance the hydrophilicity of the membrane, whereas in the treatment of wastewaters containing polystyrene microspheres (about 300 nm diameter) the membrane showed high flux (2360 L/m^2^·h or LMH) and about 99.9% separation efficiency. In another study, Alias el al. fabricated composite hollow fiber membrane for purification of oilfield wastewater. In this study, polyacrylonitrile/graphitic carbon nitride (PAN/GCN) composite nanofibers were produced using electrospinning of the solution on the alumina hollow fiber membrane collector [67]. The electrospun composite nanofibers coated the surface of the hollow fiber and increased hydrophilicity and porosity of the membrane surface. Coated nanofibers have photocatalytic activity, which can degrade the oil droplets trapped on the membrane surface under UV light and decrease membrane fouling. The combination of these two properties can dramatically improve the oil/water separation characteristic of the membrane with a low fouling problem. Furthermore, this method was adequately cost-effective and had high performance for a long period of time. The water treatment experiments were performed according to Figure 5. The results determined that the high flux (640 L/m^2^·h) and high rejection efficiency (99%) after 3 h filtration can be obtained [67].

Nanofiber composites have been also been widely investigated in many emerging desalination technologies such as membrane distillation (MD) and forward osmosis (FO). In MD, a hydrophobic membrane is used as a separation layer between a hot feed and cool permeate. The driving force is the partial vapor pressure difference brought about by temperature difference across the membrane [68,69]. MD technology is not entirely new, but it has regained much interest in the past three decades due to improvements in the module design and materials development. However, commercial uptake of MD is still limited due to inadequate membranes and relatively higher energy requirements if waste heat or renewable energy is not used. The membrane utilized for MD these days are not specifically designed for MD and, thus, they suffer from low flux and wetting problems. A suitable membrane for MD application should be hydrophobic, porous with a narrow pore size distribution, and have adequate porosity and good wetting resistance so that it can efficiently separate water and vapors [70]. Different research groups have focused on modifying membranes to enhance their efficiency using dual layering and the production of superhydrophobic, omniphobic, and Janus membranes [62,71]. Nanofibers used in MD technology are reported to have improved salt rejection and permeate flux for the desalination process [11]. However, pristine nanofiber membranes have been reported to suffer from membrane wetting due to lack of hydrophobicity, big pore sizes, and less surface functionalities. Thus many groups have attempted to improve the pristine nanofiber properties by preparing composite membrane structures based on surface modification (e.g., plasma or UV light treatment) and incorporation of nanomaterials that can provide additional functionalities [12,72,73,74,75,76]. In these methods, functional groups are added and surface roughness is enhanced to increase hydrophobicity and consequently the liquid entry pressure (LEP). For example, Li et al. [75] fabricated a Si–PVDF electrospun composite nanofiber membrane with superhydrophobic surface. The electrospinning process was performed at a voltage of 25 kV, collector speed of 500 rpm, nozzle–collector distance of 15 cm, nozzle diameter of 0.37 mm, and fluid flow rate of 5 µL/min. The produced membrane has fabulous mechanical and chemical properties and can tolerate harsh thermal, corrosion, and mechanical conditions and also showed high gas permeability through gas separation experiments. The membrane was examined in a MD setup, and due to having very low surface energy and high surface hydrophobicity, high vapor flux was obtained over a long-term duration. The combination of both mechanical and chemical resistivity and separation performance makes Si–PVDF electrospun composite nanofiber a good candidate for economic MD systems [75].

**Figure 6:**
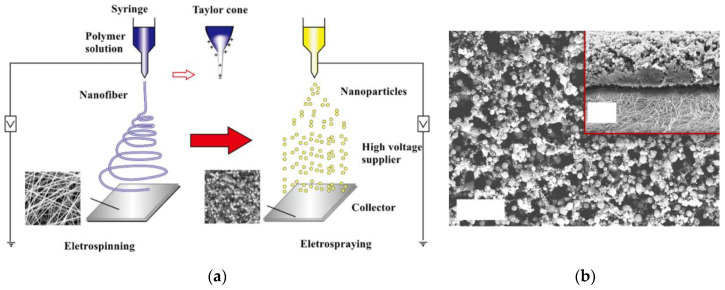
(**a**) Fabrication procedure of TiO_2_ electrospun nanofiber by the electrospinning–electrospraying process and (**b**) SEM image of the prepared membrane [79].

Deka et al. [77] fabricated a brilliant superhydrophobic nanofiber composite membrane for MD application with a water contact angle of about 170°, high surface roughness, and high flux and stability for long-period operation (7 days), and an LEP of 129 kPa in very saline water by electrospraying of aerogel–PDMS solution on the PVDF–hexafluoropropylene electrospun nanofiber membrane. In addition, different types of SiO_2_ nanoparticles were embedded in electrospun PVDF nanofiber membrane to add roughness and achieve superhydrophobic condition (contact angle > 150°) and narrow pore size distribution in the range of 1.2–1.4 µm. The membranes were used in the MD system, and results showed superb performance for water treatment process (34 LMH and 99.9% salt rejection) [78]. Guo et al. [79] fabricated a new superhydrophobic electrospun nanofiber composite membrane which has high regeneration capability and high efficiency in water treatment using MD technology. PVDF/TEOS/TiO_2_ nanofibers were first fabricated by electrospinning and then ferrate solution was sprayed on the nanofibers via the electrospraying technique (see Figure 6). The membrane was capable of decreasing membrane fouling and increasing desalination efficiency. The derived salt rejection and dissolved organic carbon removal for the fabricated membrane were over 99%. The regeneration procedure was simply performed by water flushing and experimental results revealed that the water contact angle reached to more than 150° and the membrane maintained its superhydrophobicity properties after regeneration [79].

The rough surface of nanofiber membranes provides hydrophobicity, and with some modifications, can even lead to omniphobic surfaces that are promising for treating challenging wastewaters. Omniphobic membranes are designed with re-entrant surface morphology and low surface energy and, thus, they have the ability to resist wetting from both water and low-surface-tension fluids, which is attractive for use in MD [80,81,82]. As electrospun nanofibers already possess rough surface due to their overlapping layers, many studies have utilized the nanofiber morphology and further modify them to have re-entrant structures and lower surface energy by coating process, or by incorporation of hydrophobic/modified nanomaterials, or by surface treatment. Although hydrophobicity of the liquid side of the MD membrane is a key factor for its efficiency, the other side of the direct contact MD (DCMD) membranes needs to have a hydrophilic characteristic to easily absorb water and condense water vapors [70]. Janus membranes refer to membranes with two sides having different wetting properties; that is, one side is hydrophilic, while the other side is hydrophobic. They work best in DCMD configurations [83,84]. Electrospinning has been used to fabricate Janus membranes by either wholly electrospun nanofiber configuration or by a combination of electrospinning and casting or modification processes. For the former design, hydrophobic polymer is first electrospun and then followed by hydrophilic polymer. Most often, the hydrophilicity or the hydrophobicity of both layers of nanofibers can be enhanced by either adding hydrophilic or hydrophobic nanoparticles in/on the nanofibers in the form of composites. The main issue with this Janus nanofiber composite membrane structure is the tendency of delamination as the two layers do not usually react well with each other. In one study [85], a superhydrophobic electrospun nanofiber PVDF was modified by silanized SiO_2_ nanoparticles for MD application. The other side of the membrane surface was coated with Ag nanoparticles and carboxylated MWCNTs. This resulted in a Janus membrane with superhydrophobic–hydrophilic characteristics. The modified membrane showed more than 99.8% salt rejection and its long-time fouling results halved compared to that of the neat membrane [85]. A tri-layer nanofiber composite membrane could also be fabricated to enhance the flux and fouling performance of MD.

Elmarghany et al. utilized this design and strategy to prepare porous nanocomposite with PES–CNTs as the middle layer, and both sides were coated with electrospun PVDF–HFP copolymer incorporated with CNTs (PVDF–HFP/CNTs) [86]. The results showed that compared to single PVDF–HFP/CNT layer nanofiber membrane, the DCMD performance has enhanced due to the porous middle layer [86]. Shirzad Kebria et al. [87] fabricated a hydrophobic electrospun nanofiber membrane produced from polycondensation reaction of hydroxyl groups and carboxyl groups of boehmite and nitrilotriacetic for air gap MD (AGMD) application. The modified electrospun composite nanofiber membrane increased water contact angle from 129 to 139° with water flux of 11 kg/m^2^·h and 99.9% salt rejection after a 15 h AGMD test [87]. Wang et al. [88] reported a two-layer PEI/PVDF membrane using the electrospinning method to produce an anti-oil-fouling membrane for the MD process. The membrane was crosslinked by ethanediamine (EDA), and the surface was functionalized for the anti-oil-fouling application. The oil contact angle was tested underwater, and results showed high oil contact angle (more than 140°) for the modified membrane. The experimental results showed that the adhesive force between oil and water remained very low during the extended underwater test, which explains its applicability in the treatment of oilfield wastewater using the MD method. The potential of nanofiber-based membranes and composites for MD is attractive and has been gaining much attention in the last decade. However, the long-term operation of nanofiber composite membranes is still an issue due to the relatively bigger pore sizes and wider pore size distribution. When fabricating a nanofiber membrane composite, there is a need to ensure that incorporated nanomaterials should be exposed to the surface to impart functionalities like roughness and increased hydrophobicity, but many of the studies have limitations regarding this due to the use of only a blending method. Instead, a coaxial electrospinning process could potentially help in this aspect, but more studies need to be done to ensure the robustness of the nanofiber composite membranes for long-term MD operation. Nanofiber composites have also been used for FO application usually as a porous support layer to reduce the internal concentration polarization. FO uses the natural osmotic pressure difference through a semipermeable membrane to obtain water from a dilute solution and convert it into a concentrated solution [89]. The middle layer of FO membranes needs to be highly porous for water to pass through. Compared to nanofiltration (NF) and reverse osmosis (RO), FO generally has lower energy use and fouling propensity [90]. However, it has not yet been developed for large-scale operation due to limitations in membranes as well as process parameters [89,91]. For FO, thin-film composite membranes (TFC) are mostly used because of their superior permselectivity. The active layer and support of the membranes can be separately designed, favoring improvement in the properties of the membrane [89]. A phase-inversed middle layer is commonly used, but it suffers from internal concentration polarization. To improve the structural parameters, nanofibers can be used as a middle support layer for FO membranes as this can reduce the mass transfer resistance due to their high porosity and interconnected structure. However, the main issue is the delamination potential of the active layer when synthesized on top of the rough nanofiber surface. Still, many research groups have demonstrated good FO nanofiber composite membranes taking into account the improvement in structural parameters. Pan et al. [89] prepared a PA/PAN electrospun TFC (ETFC) membrane where PAN (polyacrylonitrile) nanofiber acted as support and PA (polyamide) as the active layer, and no backing layer was used for FO–MD hybrid wastewater treatment process. The PAN laminated nanofiber support had high hydrophilicity, flexibility, and improved mechanical strength. Compared to commercial FO membranes, the PA/PAN–ETFC membrane showed an improved water flux. The results in the FO–MD hybrid system with the fabricated membrane showed a 99.8% rejection ratio which indicates the potential for efficient pollutant treatment of wastewater [89]. In another study [90], a nanofiber thin-film composite membrane (NTFC) was synthesized to improve the membrane performance during the process of FO. The NTFC was made from polysulfone (PSF), an ideal polymer with excellent mechanical, thermal, and chemical resistance and low cost. Since it is a hydrophobic polymer, a hydrophilic PAN polymer and an ultrathin polyamide layer was added to improve its hydrophilicity. The resulting PSF/PAN membrane demonstrated improved fiber structure and strength due to its multichain sheets, a desirable outcome for TFC–FO membranes. The increased hydrophilicity showed higher water flux. In general, the synthesized NTFC membrane showed a higher water flux (97.12%) than the TFC conventional membrane (95.35%), demonstrating the feasibility of the polymeric blend for FO membranes [90].

Adsorption, a surface-based process, is used to selectively adhere molecules, atoms, and ions onto a material surface usually with high surface area. It is considered the treatment choice for low concentrations of pollutants due to its low cost, easy regeneration, simple design, manageable operation, and effectiveness properties [92,93]. However, adsorbent materials have aggregation issues and, thus, immobilizing them into a support structure has been considered [92]. The high surface area of nanofibers make them good candidates for adsorption processes, either as primary adsorbent or as support structure [11]. However, pristine nanofibers usually lack functional groups for selective adsorption and, thus, many of the nanofiber-based materials for adsorption are surface-modified nanofibers or nanofiber composites. Liu et al. [94] produced electrospun composite nanofibers of PVA and PAN via a two-nozzle electrospinning method. The PVA nanofiber (thicker nanofiber) played the role of the skeleton support and PAN nanofiber (thinner nanofiber) as the functional structure. The composite membrane demonstrated a high water flux and high Cr adsorption of 133 mg/g, which is much higher compared to commercial adsorbents. In addition, grafting has increased the mechanical properties of fabricated membrane [94]. Another study utilized nanofiber composites for organic dye removal in wastewaters [95]. Electrospun composite nanofibers composed of sodium alginate (SA)were prepared, which were crosslinked with calcium chloride (CaCl_2_), glutaraldehyde vapor (GA), and trifluoroacetic acid (TFA) [96]. Using methylene blue (MB) as a model organic dye, the results demonstrated a maximum adsorption capacity of 2230 mg/g for CaCl_2_ crosslinked SA membranes and can maintain this efficiency even for five cycles. Another report [97] prepared poly(arylene ether nitrile) (PEN)/graphene oxide (GO) electrospun composite nanofibers modified by polydopamine (PDA) and used them for dye removal. The PEN supporting layer provides stability and high water flux, while the PDA–GO skin layer enhances the antifouling ability and is a successful barrier for dyes. After multirun, the resultant PEN/GO–PDA nanofiber composite membrane indicated enhanced reusability and high efficiency with low feeding pressure, exhibiting high rejection (99.8%) and permeate flux of 99.7 L/m^2^·h (0.1 mPa, pH = 3.0) for Blue 14 dye. The good thing about the nanofiber structure design is that it can easily be adapted to selectively adsorb some specific dyes while maintaining the integrity and robustness of the nanofiber membrane. The challenge in its design is on how to effectively expose the added nanoparticles on the nanofiber surface while making sure that they are also embedded strongly so as to prevent nanoparticle leakage. Homaeigohar et al. utilized a nanofiber support for immobilizing TiO_2_ nanoparticles to photocatalytically degrade dye pollutants from wastewater. The incorporation of TiO_2_ nanoparticles significantly increased the wettability of the nanofibers that led to extended contact of nanofibers and pollutants. The composite nanofiber mats obtained 95% removal of methylene blue via synergistic adsorption and photo-UV photodegradation processes.

As wastewater treatment has turned into a vital issue, the removal or elimination of heavy metals has become an important task to protect the environment and human health. Karim et al. [98] demonstrated the use of electrospun polyvinyl alcohol (PVA) and chitosan (Chi) for the removal of Pb(II) and Cd(II) ions from wastewater samples. The PVA was used to improve the poor electrospinning ability of chitosan, which is excellent for metal ion adsorption because of its polar side residues. The blend enhanced the electrospun nanofiber characteristics and uniform nanofibers membranes were produced [98]. The fabricated composite membrane showed a large number of active sites for interaction with Pb(II) and Cd(II) ions, resulting in effective adsorption of the ions. The maximum adsorption capacities for Pb(II) and Cd(II) were 266 and 148 mg/g, respectively, showing successful removal efficiency [98].

Nanofiber membranes have become an attractive option for the separation of oil from other components because of its ability to absorb and reuse. Oily wastewater damages health, ecological balance, and the environment, creating the demand for efficient technology to separate oil and water. The technologies developed to remove oil take a high amount of time, have low efficiency and high energy cost, and can cause secondary pollution. The most common treatment methods are pressure-driven separation or gravity and adsorption [99]. The second one, based on superwetting membranes, has become interesting due to its simple operation and high separation efficiency for immiscible oil/water mixtures and emulsions [99]. Nanofiber membranes have shown to be highly absorbent especially with their high surface area, successfully separating oil from water in a short time while having a recycling potential [11]. Fouling, caused by the adsorption of the oil, is the main problem in oil/water emulsion separation, which blocks the pores and decreases the flux [95]. Li et al. (2018) prepared electrospun nanocomposite nanofibers of poly(vinyl alcohol-co-ethylene) (PVA-co-PE)/SiO_2_ nanofiber with superhydrophilic and oleophobic characteristics coated on the polyamide/PVA-co-PE nanofibers as substrate. The fabricated composite nanofibers were tested for oil-in-water emulsion and oil/water separation. Nanofiber addition increased the ability of SiO_2_ nanoparticles to form film and bonds. In addition, the nanofiber composite-coated membrane has the benefit of environmental friendliness, lower cost, and high throughput [99]. Furthermore, the surface of produced membranes was observed to be uniform and continuous. From the various oil/water mixture separation by gravity, the membrane demonstrated high separation performance. The results, in general, indicate good oil/water separation performance of coated composite membranes, suggesting potential application for industrial oil/water separation [99]. Wang et al. [93] prepared TiO_2_-coated PAN nanofiber membrane. The acquired TiO_2_/PAN–Si electrospun composite nanofiber demonstrated a good superoleophobicity, superhydrophilicity, and self-cleaning properties for application in the separation of oil/water emulsions. The emulsion separation performance was evaluated, and the results demonstrated that the drop size and oil type have a direct influence on the separation performance. Fouling was found in the separation of soybean oil because of the smaller oil drop size; nonetheless, it can be eradicated by UV irradiation. After five cycles, the TiO_2_/PAN–Si membrane flux can be fully recovered, showing good performance with the highest flux of 200,000 L·m^−2^·h^−1^·bar^−1^ [93]. Table 1 shows a summary of various nanofiber composites used for membrane separation and water purification.

#### 4.1.2. Air Filtration

Air pollution is a major issue for many countries in the world. The presence of particulates, volatile organic compounds (VOCs), mineral powder, carbon, bacteria, etc., in the atmosphere can be inhaled by people and endanger their health; thus, it is necessary to filter out these pollutants [11,127]. Using nanofiber membranes as filter materials has some advantages compared to other filters made of activated carbon and fiberglass. Nanofibers have high surface to volume ratio, low resistance, and controllable size, resulting in high performance for permeability and selectivity. The high surface area also provides many active sites for decorating with functional materials that could enhance the overall affinity of the membrane to filter pollutants [127]. However, due to the ultrafine structure of nanofibers and small pore sizes, there is a challenge in the big pressure drop which can affect the filtration performance for long term operation. Some groups utilized a multilevel structured approach to lessen the pressure drop but still maintain high filtration efficiency. Electrospun nanofibers can also be decorated with materials that can provide antibacterial properties. In a previous study, Bortolassi et al. [142] explored the design of uniform PAN nanofiber filter decorated with Ag nanoparticles. The resultant nanofiber composite filter demonstrated low pressure drop, thereby allowing the air to pass through easily. The addition of 1 wt% AgNO_3_ in PAN solution was enough for the filtration membrane to demonstrate great antibacterial activity and filtration efficiency. This report suggests that Ag/PAN nanofiber membranes can be used for wide air filtration applications, even in large-scale production [142]. Huang et al. [143] fabricated poly(ε-caprolactone)/polyethylene oxide (PCL/PEO) electrospun composite nanofiber using electrospinning and solvent vapor annealing (SVA) methods. SVA was used to treat the surface of the nanofibers and improved the efficiency of the nanofibers for capturing PM2.5 aerosols from highly polluted test conditions (80% removal efficiency). High-porosity (96%) Ce–W–TiO_2_/PVA electrospun composite nanofibers were successfully fabricated using the electrospinning–calcination method for NOx–SCR catalytic reactions. This reaction is performed to prevent pollutant gases, which are formed during car combustion process, from entering the air. The catalytic performance of the nanofibers was analyzed and results showed that due to high porosity and unique morphological structure, the nanofiber composites were stable (>120 h) with high reusability [144]. Table 2 details some studies utilizing nanofiber composite membranes for air filtration application.

### 4.2. Biomedical and Healthcare Applications

Electrospun nanofibers have been widely investigated in the field of biomedical and tissue engineering due to their interesting properties and versatility to be designed into various morphologies, such as nonwoven form, aligned nanofibers, core–shell structure, and hybrid nanocomposites. In tissue engineering, scaffolds are used to supply support to regenerate damaged tissues. The unique characteristics of electrospun nanofibers, such as loose structure, high porosity, and superb flexibility can mimic the extracellular matrix (ECM) for cells to grow and, thus, they have attracted great attention for use in tissue engineering. Moreover, most polymers―including biodegradable and biocompatible materials―can be electrospun, which is perfect for application [11]. Generally, pristine nanofibers have the needed morphology and structure for tissue engineering, but lack functionalities that can enhance cell growth and, thus, many of the investigated nanofiber scaffolds have incorporated nanomaterials such as carbon-based materials, ceramic (like hydroxyapatite), and some drugs [28,153,154]. 3D nanofiber scaffolds provide the conditions for better attachment of single cells and tissues as well as for cell–cell interactions, which is one of the most important parameters for regulating cell cycles and functions in tissue engineering. Some strategies for 3D nanofiber scaffold fabrication include multilayer electrospinning, folding, or stacking 2D fiber films using a 3D collecting template or self-assembly [28]. Composite nanofiber scaffolds containing materials similar to the mineral component of natural bone, like hydroxyapatite, are also being used in bone surgeries to mimic natural bone regeneration. Composite nanofibers with collagen have been used for skin trauma to improve cell attachment and proliferation. The electrospun nanofibers can also be used for delivery of drugs at a controlled rate for accelerating tissue regeneration or having antibacterial influence. In this way, the desired material is slowly released to have a long-term and uniform influence on the tissue.

Nanofibers can be used as substrate material for some biomaterials that are quickly degraded. For example, gelatin, which is a congener protein of collagen, has been used as biomaterial but has fast degradation, and its high hydrophilic surface makes it inappropriate as the base material [155]. Thus, a material such as polycaprolactone (PCL) can be used to serve as a base material for gelatin. Ren et al. prepared electrospun PCL/gelatin hybrid nanofiber scaffold for potential application as guided bone regeneration (GBR) membranes. The use of gelatin in the membrane showed improved cell viability and wettability for better proliferation and cell adhesion. The tested nanofiber membranes showed cell viability (>80%), indicating nontoxicity and supporting cell proliferation, making it a promising candidate for GBR membranes. Also, the two macromolecules (PCL/gelatin) are easy to get, cheap and biomedically safe [155]. Facile electrospinning fabrication can also be used to add some drugs that can be designed for controlled release. Rezk et al. [156] fabricated a composite nanofiber scaffold made of poly(vinyl alcohol)–poly(vinyl acetate) (PVA–PVAc) and loaded with simvastatin superficial layer to get an improved osteogenesis process through the continuous release of the drug [156]. PVA is biologically friendly and has elasticity, flexibility, proper mechanical properties, nontoxicity, swelling ability, and biodegradability. However, its instability in water limits its use in drug delivery applications. Therefore, PVA was crosslinked with biocompatible and biodegradable PVAc having hydrolysable groups. A simvastatin drug was loaded into the blended solution of PVA–PVAc to promote the regeneration of bone [156] and results showed good bioactivity, inducing the precipitation of bone-like apatite minerals on its surface and successfully simulating physiological conditions for cell growth [156]. Carbon-based nanomaterials have also been used as filler materials for nanofiber scaffolds such as graphene oxide. Nalvuran et al. used reduced graphene oxide (RGO) (0.5%, 1.0%, and 2.0%) and silk fibroin (SF) on a composite nanofiber membrane for evaluating its potential in tissue regeneration. Incorporated RGO promoted thermal and mechanical stability in the membrane while silk fibroin (SF), a biopolymer, provides biocompatibility, morphologic flexibility, permeability, and biodegradability [157]. In addition, the produced composite (RGO-SF) nanofibers showed better cell viability and were hemocompatible [157]. Cellular studies revealed potential of composites to reinforce spreading, proliferation, and attachment of cells, mostly due to the surface topography [158].

Electrospun nanofibrous dressings have high surface-to-volume ratio, allow gas permeation, help to regulate wound moisture, enhance tissue regeneration, promote removal of exudates, and have high porosity, which makes them ideal for use in wound healing. Previous studies have shown low inflammatory reaction and fast re-epithelization with the use of nanofiber-based wound dressing [11]. Nanofibers have demonstrated different possibilities for drug delivery applications as they can be structurally designed to contain drug molecules and release them in a controlled way. The mechanism for drug release is related to the drug desorption from the nanofiber surface and subsequent diffusion in the pores of the web [11,159]. Their small pores also help lower infection by limiting microorganism entrance to the wound [160]. Various types of polymers have been investigated to analyze their applicability for wound dressing. Poly(lactide-co-glycolide) (PLGA) is a biocompatible and biodegradable polymer that is available in a wide molecular weight range and has recently been used in some clinical and research activities as a carrier for drug delivery purposes. Fabrication of antimicrobial drug-loaded PLGA-based electrospun nanofiber is a promising material for wound-dressing applications [160].

Garcia-Orue et al. [160] prepared an electrospun composite nanofibrous membrane using PLGA aloe vera (AV) extract with lipid nanoparticles (NLC or nanostructured lipid carriers). Aloe vera has been used for wound healing due to its antifungal, anti-inflammatory, hypoglycemic, and antibacterial properties which prevent wound infection. As for NLCs, they were added to the PLGA/AV formulation to incorporate lipid content that avoids adhesion to the wound [160]. The nanofiber composite dressings demonstrated similar results for effective wound healing, which indicates a promising strategy for chronic wound treatment [160]. Microbial growth affects the healing rate in a wound and is altered by the environment of wound beds. As an important aspect, wound dressings need to last longer before replacement and need to heal wounds rapidly. Bacterial infection delays the wound-healing process and, therefore, antimicrobial agents for wound dressings are being demanded [161]. Jaganathan et al. [162] used polyurethane (PU) polymer and copper sulfate as an antimicrobial agent to fabricate an antimicrobial nanofiber wound dressing. PU is commonly used due to its oxygen permeability, good barrier properties, that that it has tunable chemical and excellent mechanical properties, good biocompatibility, and can be designed to degrade in a biological environment. Properties such as mechanical strength, swelling ratio, and cell adhesion, that aid in wound-healing application, have made PU a good candidate for such applications. Copper is a chemically stable, low-toxicity metal having antimicrobial properties [162]. The electrospun PU–copper sulfate nanocomposite demonstrated a hydrophilic nature favoring wettability, proliferation, and fibroblast adhesion for new tissue growth. However, the nanocomposites had smaller fibers and pore diameter in comparison to pure PU. Copper sulfate addition increased the mechanical strength and surface roughness. Coagulation assays showed blood compatibility by demonstrating extended blood clotting time compared with pure PU. A less toxic nature for PU–copper sulfate membrane was demonstrated through its enhanced cell viability and low hemolytic index compared with the PU membrane [162].

**Figure 7:**
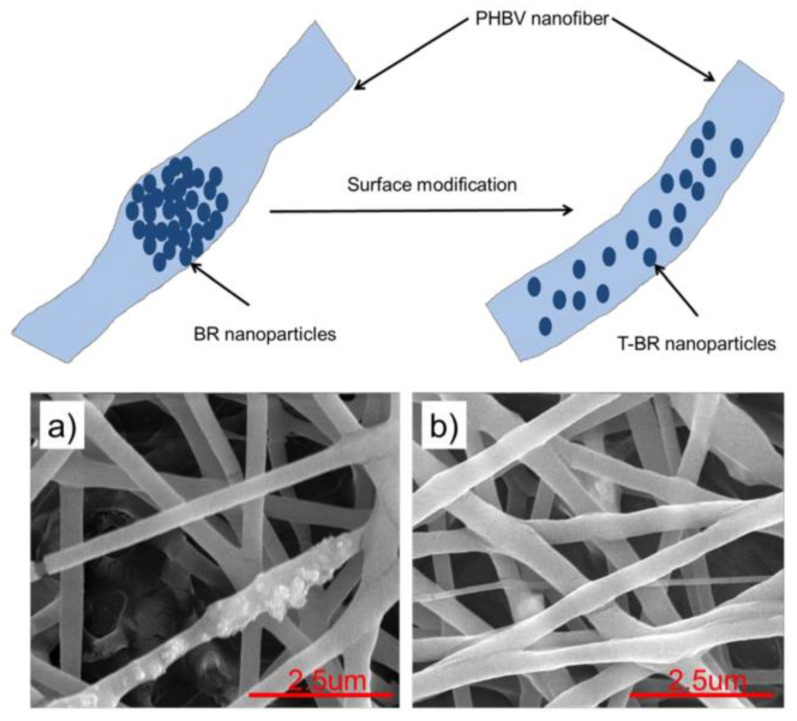
Schematic illustration and SEM images of PHBV nanofibers containing 15% of (**a**) bredigite (BR) and (**b**) T-BR nanoparticles [163].

Bredigite polymer electrospun nanofibers have been widely studied to investigate their applicability in wound-dressing activities. It has been used as a scaffold in some studies, but results revealed that while the bioactivity of the composite nanofibers was improved, and the low dispersibility and high agglomeration of nanoparticles decrease the efficiency of prepared electrospun nanofibers [163]. In another attempt, bredigite (BR) nanoparticles have been modified by an organosilane coupling agent to increase its dispersibility [163]. The SEM results reveal that the modified BR nanoparticles are widely dispersed in the body of the nanofibers without any agglomeration (Figure 7). Moreover, the mechanical and biodegradation rate of the scaffolds dramatically improved after BR modification.

The use of composite nanofibers has demonstrated great potential for biomedical applications. Nonetheless, effective elaboration of well-blended composite fibers is a great challenge at the molecular level because of poor miscibility between polymers and ceramic particles, ending up in a poorly blended composite with weak mechanical strength and incontrollable material properties [164]. Chitosan (CS) and polycaprolactone (PCL) blends have shown biocompatibility, stability, and mechanical strength in biomedical studies, representing ECM tissue with an excellent framework for proliferation, differentiation, and adhesion. Chitosan has versatile biological characteristics, while PCL has mechanical but lacks cell recognition sites and affinity due to its hydrophobicity. By appropriately blending, PCL–CS/MgO composites have the benefit of incorporating the favorable aspects of PCL with CS without needing chemical crosslinking to maintain their desirable mechanical properties and structure [164]. A summary of the various nanofiber composites for biomedical and healthcare sectors is listed in Table 3.

### 4.3. Energy and Sensor Application

The energy sector has, for years, been investigating and improving its technology in order to provide cleaner and cost-effective energy. Nanofibers are part of the improvement of the different energy technologies. Nanofiber membranes have been used as catalyst to provide high activity, high durability, and high poisoning resistance for fuel cells and can be utilized as electrodes for lithium ion batteries to improve their capacity and performance [11].

**Figure 8:**
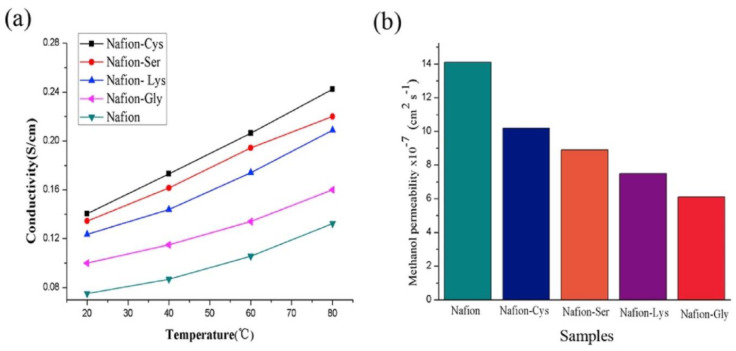
(**a**) Conductivity and (**b**) permeability (methanol) results of various nanofiber composites [194].

For improvements of proton exchange membrane fuel cells (PEMFCs), it is crucial to develop an ionomer membrane that attains high proton conductivity and low permeability for gas while maintaining high thermal, chemical, and mechanical stability, low deformation, and low cost. Due to the high surface area and controllable characteristics, nanofibers can be used as proton exchange membranes where filler materials are added or can be hybridized with other materials to provide the needed functionalities of the membrane. Perfluorosulfonic acid membranes (Nafion series) were usually used as PEMs. However, their high cost, serious proton conductivity reduction at high temperatures, high methanol permeability, and poor dimensional stability are reasons for considering other alternatives [194]. Wang et al. used electrospun SiO_2_ nanofibers decorated with biofunctional amino acid molecule chains (cysteine, serine, lysine, and glycine) to build efficient proton-conducting amino acid channels. [194]. The overall results showed significant improvement of the composite membranes as PEMs in dimensional and thermal stability, methanol permeability, water uptake, and proton conductivity. The best proton conductivity was shown by the Nafion–cysteine membrane, while the best methanol permeability and dimensional stability was demonstrated by Nafion–glycine. However, Nafion–glycine also showed the lowest proton conductivity and water uptake due to having the lowest number of hydrophilic groups among the amino acids used (see Figure 8) [194].

Lithium-ion batteries (LIBs) are used for energy storage in many portable electronic devices like digital cameras, laptops, mobile phones, and communication equipment due to their high operational voltage, high energy density, low self-discharge rate, and long cycling life. The separators in batteries are used to prevent physical contact between the cathode and anode and maintain liquid electrolyte for rapid transport of ions, important to the battery’s safety [195]. Polyolefin microporous membranes are usually used as Li-ion separators but suffer from disadvantages such as low porosity, poor electrolyte wettability, and inferior thermal stability, limiting the performance of the battery. Aromatic polyimides (PIs) have been proposed as alternative material as they possess great chemical resistance, superior mechanical properties, and good thermal stability. Kong et al. [195] prepared nanofiber membrane using fluorinated polyimide (FPI) which showed some advantages compared to its non-fluorinated analogue, such as high polarity that enables greater affinity toward polar liquid electrolyte. The thermocrosslinking procedure for FPI regulates the pore sizes and improves mechanical strength and gives outstanding electrolyte uptake and thermal stability. On top of that, the battery fabricated with FPI separators showed remarkable cycle performance and rate capacity with the benefit of preventing penetration and growth of dendritic lithium.

Generally, polymer electrolytes are made of solid polymer electrolytes (SPEs) and gel polymer electrolytes (GPEs). GPEs can result in short circuit, explosion, overheating, and insufficient capacity due to the polymer meltdown at high temperature. To reduce the risk and avoid this problem, polymer skeletons must have good thermal stability [196]. Wang et al. (2018) improved GPE performance by electrospinning a PEI nanofiber membrane incorporating halloysite nanotubes (HNTs) [196]. The HNT/PEI composite nanofiber GPE membranes showed high ionic conductivity, good thermal stability, good affinity between the electrolyte and the nanofibers, low interfacial resistance, and superior electrode and electrochemical performance while fulfilling requirements of high-performance batteries. However, interfacial resistance seemed to increase because of the agglomeration of HNTs caused by the hydroxyl groups in the membrane surface [197].

The unique properties of electrospun nanofibers, like electrochemical activity and large surface area, make them highly desirable for use in electrochemical energy devices such as supercapacitors. Many materials such as carbon, metal sulfides, and nanocomposites have been utilized in the form of nanofibers as electrodes for supercapacitors. They are also prepared in various architectures such solid, core–sheath, or hollow designs. For example, carbon nanofiber electrodes can be prepared by carbonizing polymer precursors such as PAN and polyimide at high temperature under an inert atmosphere. A wide variety of nanofiber designs and morphologies have been tested and promising results as supercapacitor electrodes have been obtained. However, further tuning of the properties of the nanofibers, such as architecture and morphology as well as composition optimization, is still needed to enhance their overall performance. 3D nanofiber structure is said to be a good design that enables a shorter diffusion pathway for the electrolytes. One is referred to a comprehensive review by Lu et al. [198] of the potential of electrospun nanofibers and their challenges for supercapacitor applications.

Flexible and wearable electronics are currently in high demand because of their foldability and stretchability. Energy devices that are flexible can store or convert energy by bending repetitions, folding or stretching without loss of performance, with high potential in wearable and portable electronics like touch screens, wearable sensors, military garment devices, and biomedical devices [199]. Electric power generators that are power-based are highly desirable because of their comfort and light to wear. The devices can be designed for low strain levels, soft fiber, and high fatigue resistance. Piezoelectric nanomaterials can be integrated with composite polymer and fibers. However, the challenge is the development of a durable and flexible electrode for electric power wearable generators produced by the kinetic energy of human motions [199]. As an example, a nanogenerator with silver-coated polyamide electrodes incorporated with piezoelectric fabric of NaNbO_3_ nanowires and PVDF composite nanofibers was “sandwiched” and survived without failure after at least 1,000,000 compression cycles. The randomly oriented electrospun nanofiber membrane can be used without extra poling treatment in a piezoelectric power generator to generate several volts [199]. Bairagi et al. [200] fabricated flexible nanogenerators from solution melt of PVDF polymer and 4 wt% KNN nanorods using an electrospinning technique. The prepared nanogenerator showed high dielectric constant of 175. This generator has capability for production of 3.7 V and 0.326 µA. These values are adequate for using in portable electronic gadgets as high-efficiency power sources.

For sensor applications, electrospun nanofibers have been used due to the flexibility in material selection, ease in incorporating active agents, and the possession of high surface area to volume ratio. Sensors of this type can be more sensitive than electronic sensors depending on the selected material [199]. Textile sensor polymer-based have the advantage of being adaptable to shape and surface in addition to being portable. The incorporation of nanoparticles in/on the nanofiber is one of the main techniques to enhance the sensor capability of the composite material. Guan et al. [201] utilized cholesteric liquid crystal (CLC) nanoparticles immobilized in polyvinylpyrrolidone (PVP) nanofibers to provide temperature-response properties. Results indicated good temperature response between 25 and 60 °C, giving them promise for use in applications as optical and thermal sensors. Another way is through the production of piezoelectric nanofibers. In a study by Chinnappan et al., different piezoelectric electrospun nanofibrous materials for the production of self-powering wearable electronic textiles have been reported. They designed a one-step nanogenerator based on PVDF. They evaluated the piezoelectric properties of the fabricated composites with results showing improvement in piezoelectric properties of nanofibers by the induced change in crystalline structures and show they can be used as wearable electronic textiles. In addition, incorporating the nanofiber layer with electrodes within the structure improved the output of electricity by 1 volt [199]. Andre et al. [202] produced a novel electrospun ceramic nanofiber sensor for detection of very low concentrations of NH_3_ (44 ppb in a response time of 17 s) with high accuracy with respect to other nitrogenated compounds from the combination of IN_2_O_3_-reduced graphene solution mixture. Metal oxides fibers are widely used as gas sensing materials due to their electrical, thermal, and mechanical properties. The accuracy of the sensors directly depends on the morphology and available surface area of the fibers. Therefore, electrospun nanofibers can bring a tremendous opportunity to improve the gas sensing capacity of the metal oxides by preparing of 1D nanofibers having very high aspect ratio and large-scale and cost-effective production capabilities. Khalil et al. have fabricated electrospun nickel oxide nanofibers for using in the gas sensing industry. Results demonstrated that the special morphology of electrospun nanofibers leads to faster and more complete resistance recovery [203]. SnO_2_ electrospun nanofibers were also fabricated for hydrogen gas sensing at low temperatures and obtained the highest response, resulting in favorable accuracy for working in low-temperature environments [204].

## 5. Conclusions and Outlook

The potential of electrospun nanofiber composites has been highlighted in this review, showing many applications of this structure in different fields. The facile fabrication, high surface area, and controllable morphology and structure of electrospun nanofibers provide vast opportunities for functionalization, incorporation of fillers, and modification to enhance the overall properties of the nanocomposites. There have been enormous developments in the fabrication, modification, and applications of electrospun nanofiber composites; however, most of these have been in the academic aspect while they have still not widely adopted for commercial and large-scale applications [10]. Nonetheless, the potential of nanofiber composites is still enormous, with many less explored applications in agriculture and food packaging and formulation [22]. Nanofiber composites have the flexibility to enhance or limit nanofiber properties to either improve performance or achieve a specific activity. However, there are still various challenges that need to be addressed to fully scale up the nanofiber composites. Nanofibers in the biomedical field have been widely investigated, and new directions are now on the incorporation of drugs for controlled drug delivery or coating with nanoparticles as antibacterial nanofiber coating or for photocatalysis. The main challenge, though, is how to make sure that filler materials are exposed to the surroundings while maintaining a firm hold on the nanofiber substrate to avoid dislodging. There has also been exponential growth in the number of research studies for water-related applications, especially those utilizing membrane separation processes. However, still, the issue of surface chemistry and low mechanical strength are ongoing challenges that need to be addressed. When nanofibers are used as support layer, there is an issue of potential delamination of the top active layer due to the rough surface of the nanofiber and the difference in material properties. The big and wide pores of nanofiber composite membrane may also lead to wetting issues in membrane distillation. The energy generation and storage device field has also seen rising uptake of nanofiber composites, and it has shown great strides in performance improvements. However, enhancement in nanofiber architecture design and composition optimization are still needed.

Perhaps the greatest challenge still faced by nanofiber composites is the mass production capacity. There are many companies that offer lab-scale setup for electrospinning, which can produce sample sizes from A4 paper size to even in excess of 1 m^2^ size. However, the industrial upscaling of nanofibers, especially in their nanocomposite forms, is rather limited and still in the development stage, although some companies like Elmarco (https://www.elmarco.com/) and Fnm Co. (http://en.fnm.ir/) can supply industrial-level capacity electrospinning devices with some modifications from the conventional single-needle spinneret design. Muti-spinneret electrospinning for mass production may be good, but it can undergo unstable jets due to the proximity of jets, leading to non-uniform fibers. Rotating drums or needleless spinning could be used but may suffer from non-uniform fibers especially for nanofiber composites where filler nanoparticles or functionalization steps are added. Most of the industrial applications are for air filtration, including nanofiber facial masks, but high-temperature nanofiber filters and battery separators have also been reported to have large production capacity (~2000 m^2^/day) [205]. Some companies in the biomedical field such as Nicast (http://nicast.com/) and Xeltis (http://xeltis.com/) have also started using electrospun nanofibers. Nicast has fabricated a vascular access for hemodialysis using nanofibers while Xeltis used electrospun nanofibers for blood vessel or heart valve fabrication for cardiovascular restoration. The continuous development of new electrospinning devices with high throughput will eventually lead to the mass production of nanofiber composites in the future.

## Figures and Tables

**Table 1:** Nanofiber composite membranes used for membrane separation and water purification applications.

Main Substrate	Modification	Method	Application	Ref.
PH	PFTES-TiO_2_	Coating	MD	[100]
PVDF/PES		Three layer	DCMD	[71]
PA/PAN		Supported TFC	FO–MD	[89]
PSF/PAN			FO	[90]
PAN	TiO_2_		Oil/water separation	[95]
PVA-co-PE	SiO_2_	Supported	Oil/water separation	[99]
Chitosan/PVA	Zeolite	Incorporation	Heavy metal adsorption	[92]
PEN	GO/PDA	Incorporation	Adsorption	[97]
PVA	Chitosan		Adsorption	[98]
MgAl-EDTA	LDH–PAN	Intercalation	Adsorption	[101]
PAN	Ag, CuO, ZnO	Incorporation	Antibacterial	[102]
ATP	GO	Intercalating	Molecular separation	[103]
PSF/PVDF		Dual layer	MD	[104]
PH	TFCS–TiO_2_	Incorporation	MD	[105]
PH/PAN		Dual layer	MD	[106]
PSU	PDMS	Coating	MD	[107]
PI	FAS–SiO_2_	Coating	MD	[108]
PP/PVA		Dual layer	MD	[109]
PH	FTES–CNTs	Incorporation	MD	[110]
PH	FTES–CNTs	Incorporation	MD	[111]
PH	CNTs	Incorporation	MD	[112]
PVDF	MOF	Higher	MD	[113]
PH–FTES	CNT	Incorporation	MD	[114]
SiO_2_	SiO_2_	Incorporation	MD	[115]
PH	PDMS	Coating	MD	[116]
PH	PVDF–PDMS	Coating	MD	[117]
PVDF	SiO_2_	Incorporation	MD	[118]
FTES–TiO_2_	PH		MD	[119]
SBS		Single needle	MD	[120]
PS		Single needle	MD	[121]
PVDF	FTCS–TiO_2_	Coating	MD	[122]
PEI	PMO	Incorporation	MD	[123]
PH	PVDF	Coating	MD	[124]
PVDF	SiO_2_	Incorporation	MD	[75]
PH	FAS	Coating	MD	[125]
PAN–CNT	TiO_2_–NH_2_	Incorporation	Photocatalytic	[126]
PAA		Incorporation	Adsorption	[127]
TiO_2_	SiO_2_	Incorporation	Photocatalytic	[128]
Nylon 6	PPy	Incorporation	Adsorption	[129]
PAN–CNT	TiO_2_–NH_2_	Incorporation	Adsorption	[130]
ZnO	CuO	Incorporation	Photocatalytic	[131]
PAN	GO	Incorporation	MF	[132]
Chitosan	PMMA	Incorporation	MF	[133]
PAN	PVA–glutaraldehyde	Two nozzle	MF	[134]
PES/CNT	PcH/CNTs	Triple layer	MD	[86]
Polystyrene		Electroblowing technique	MD	[135]
PVDF−HFP	PDMS/PVDF	Spin–spray	MD	[77]
PVDF	PTFE		MD	[136]
PVDF	Polypropylene	Facile vacuum filtration method	MD	[137]
PVDF	Polyester		MD	[99]
PVDF	SIO_2_		MD	[78]
PVDF–SiO_2_	Ag–MWNT	Coating	MD	[85]
PTFE/PEO		Hollow fiber coating	MD	[138]
PVDF	HB–Den–NTN	Polycondensation	MD	[87]
Polysulfone		Heat post-treatment	MF	[64]
PVDF	TiO_2_	Spin–spray–DAF	MD	[79]
PVDF	PDMS	Facile dip coating	VMD	[139]
PVDF	GO	Incorporation	VMD	[140]
YSZ/Silica		Sol–gel	MF	[141]

**Table 2:** Various electrospun nanofibers used in air filtration.

Main Substrate	Modification	Fiber Diameter (nm)	Porosity %	Remarks	Ref.
PLA		150–300	87	Low pressure drop	[145]
PAN	Ag	250	96	Excellent antibacterial activity	[60]
				High NaCl removal efficiency	
				Wide range of particle filtration	
PAN		200		High PM2.5 removal efficiency	[146]
PA-6		150		High PM2.5 removal efficiency	[147]
PU		120		High PM2.5 removal efficiency	[148]
PA-6	PAN	272		High filtration efficiency	[149]
PVC	PU	960	10	High filtration efficiency	[150]
PAN	PU	175	83	Superhydrophobic	[151]
PAN	SiO_2_	600	70	High filtration efficiency	[152]
PCL	PEO	2000		High mechanical stability	[143]

**Table 3:** Applications of modified and composite nanofibers in biomedical and healthcare sector.

Main Substrate	Modification	Application	Remarks	Ref.
Silk	PEO	Scaffolds	+Good performance	[165]
			+High strength	
PCL	Gelatin	Guided bone regeneration	+High wettability	[155]
			+Nontoxic	
			+Cheap	
PVA/PVAc	PCL–CA–TCP	Bone regeneration and drug release	+Biocompatible	[156]
			+High cell attachment	
			−Low wettability	
RGO	Silk fibroin	Tissue regeneration	+Supported cell viability	[157]
			+Hemocompatible	
			−Loss of mass at 100C	
PU	Graphene	Tissue regeneration	+Electroconductivity	[158]
			+Nontoxic	
			+High mechanical property	
PLGA	Nanostructured lipid carriers	Wound dressing	+High wound healing	[160]
			+Easy handling	
Chitosan	Silver and cinnamaldehyde	Wound dressing	+Improved antimicrobial activity	[161]
			+Noncytotoxic behavior	
			−Low stability	
Chitosan–PU	Silver	Dental barrier membranes	+Biocompatible	[166]
			+Antibacterial	
			+Cheap	
PVA	ZnO	Medical gown	+High strength	[167]
			+Self-cleaning	
			+ Blocking of UV and bacteria	
MgO	PCL–CS	Biomedical	+Good cell viability	[164]
			+Cheap	
			+Toxicity at high pH	
PLA–poly(butylene carbonate	GO	Antibacterial applications	+High antibacterial performance	[168]
			+Uniform GO distribution	
Chitosan	PEO/silica	Bone regeneration	+High biocompatibility	[169]
			+Cytocompatible in bone-forming	
Potato starch	PVA	Wound healing	+Promoted fibroblast cellular proliferation	[170]
			+good wound healing	
PVA	CNT–AgNP	Wound healing	+Durable antibacterial activities	[171]
PVA	NiO/ZrO_2_	Bone tissue engineering	+High aspect ratio	[172]
			+Green processing	
			+Dental and bone tissue application	
PMMA	-	Active packaging	+High good water resistance	[173]
			+Nontoxic fabrication	
PLGA		Pharmaceutical industries	+Good in vivo results	[174]
			+High crossing efficiencies	
PCL		Drug delivery	+High drug loading	[175]
			+Long-time drug release	
PLLA		Regenerative medicine	+High bone regeneration	[176]
			+Sufficient water solubility	
Gelatin		Tissue regeneration	+Precise mapping	[177]
			+Efficient self-powered	
Starch		Tissue engineering	+Water sensitive	[178]
			+High mechanical properties	
Collagen		Tissue engineering	+Favorable crosslinking	[179]
			+Structurally stable	
PLGA-curcumin		Drug delivery	+Wound-healing activity	[180]
			+Antioxidant and anti-inflammatory properties	
PCL–chitosan		Tissue engineering	+Excellent cellular infiltration	[181]
			+No calcification or aneurysm	
PHBV–gelatin		Tissue engineering	+Useful carrier for tissue engineering	[182]
			+Milieu supporting	
Hydroxyapatite		Tissue engineering	+Effectively supported proliferation of MG-63	[183]
			+Promoted biomineralization	
			+High mechanical strength	
PVA/alginate–bioglass		Tissue engineering	+High hydrophobicity	[184]
			+High porosity	
Polyurethane (PU)–dextran–estradiol		Wound dressing	+Proper skin regeneration	[185]
PLGA–tussah silk–grapheme oxide		Drug delivery and tissue engineering	+Accelerated mesenchymal stem cells differentiation	[186]
			+Improved mechanical properties	
Polycaprolactone	Hydroxyapatite	Scaffolds	+Good osteoblast activity	[187]
			+Good osteoblast viability	
Eggshell	Hydroxyapatite and poly(lactic) acid	Tissue scaffold	+Increased thermal properties	[188]
			+High mechanical strength	
Polycaprolactone	Hydroxyapatite and rifampicin	Drug	+Good cytocompatibility	[189]
			+Enhanced antibacterial property	
PAN	ZnO–Ag	Antibacterial	+Simple and cost-effective method	[60]
			+High antibacterial functionality	
PHBV	Bredigite	Bone tissue engineering	+Improved mechanical performance	[163]
			+Improved bioactivity	
			+Appropriate for bone tissue engineering	
PCL	TiO_2_	Antimicrobial	+Superior antibacterial property	[190]
			+Good bioactive properties	
PCL/chitosan	Sr–CaP	Bone regeneration	+Higher ALP activity level	[191]
			+Better matrix mineralization	
PHB–CTS	Alumina	Bone tissue engineering	+High tensile strength	[192]
			+Hydrophilicity and surface roughness	
PVP	Silicon oxycarbide-doped Ag	Antibacterial activity	+Good antibacterial activity	[193]
			+Suitable permeability	

## References

[1] Ahmed F.E., Lalia B.S., Hashaikeh R. (2015). A review on electrospinning for membrane fabrication: Challenges and applications. Desalination.

[2] Araki J., Miyayama M. (2020). Wet spinning of cellulose nanowhiskers; fiber yarns obtained only from colloidal cellulose crystals. Polymer.

[3] Wu S., Zhou R., Zhou F., Streubel P.N., Chen S., Duan B. (2020). Electrospun thymosin Beta-4 loaded PLGA/PLA nanofiber/microfiber hybrid yarns for tendon tissue engineering application. Mater. Sci. Eng. C.

[4] Liu Y.J., Tan J., Yu S.Y., Yousefzadeh M., Lyu T.T., Jiao Z.W., Li H.Y., Ramakrishna S. (2020). High-efficiency preparation of polypropylene nanofiber by melt differential centrifugal electrospinning. J. Appl. Polym. Sci..

[5] Kamin Z., Abdulrahim N., Misson M., Chiam C., Sarbatly R., Krishnaiah D., Bono A. (2020). Use of melt blown polypropylene nanofiber templates to obtain homogenous pore channels in glycidyl methacrylate/ethyl dimethacrylate-based monoliths. Chem. Eng. Commun..

[6] Tan N.P.B., Paclijan S.S., Ali H.N.M., Hallazgo C.M.J.S., Lopez C.J.F., Ebora Y.C. (2019). Solution Blow Spinning (SBS) Nanofibers for Composite Air Filter Masks. ACS Appl. Nano Mater..

[7] Bazrafshan V., Saeidi A., Mousavi A. (2020). The effect of different process parameters on polyamide 66 nano fiber by force spinning method. AIP Conference Proceedings.

[8] Hou Y., Cheng L., Zhang Y., Yang Y., Deng C., Yang Z., Chen Q., Wang P., Zheng L. (2017). Electrospinning of Fe/SiC Hybrid Fibers for Highly Efficient Microwave Absorption. ACS Appl Mater. Interfaces.

[9] Aruchamy K., Mahto A., Nataraj S.K. (2018). Electrospun nanofibers, nanocomposites and characterization of art: Insight on establishing fibers as product. Nano-Struct. Nano-Objects.

[10] Lim C.T. (2017). Nanofiber technology: Current status and emerging developments. Prog. Polym. Sci..

[11] Tijing L.D., Woo Y.C., Yao M., Ren J., Shon H.K. (2017). 1.16 Electrospinning for Membrane Fabrication: Strategies and Applications. Comprehensive Membrane Science and Engineering.

[12] Tijing L.D., Choi J.-S., Lee S., Kim S.-H., Shon H.K. (2014). Recent progress of membrane distillation using electrospun nanofibrous membrane. J. Membr. Sci..

[13] Pant H.R., Pant B., Pokharel P., Kim H.J., Tijing L.D., Park C.H., Kim H.Y., Kim C.S. (2013). Photocatalytic TiO_2_–RGO/nylon-6 spider-wave-like nano-nets via electrospinning and hydrothermal treatment. J. Membr. Sci..

[14] Tijing L.D., Ruelo M.T.G., Amarjargal A., Pant H.R., Park C.-H., Kim D.W., Kim C.S. (2012). Antibacterial and superhydrophilic electrospun polyurethane nanocomposite fibers containing tourmaline nanoparticles. Chem. Eng. J..

[15] Sekar A.D., Manickam M. (2019). Current Trends of Electrospun Nanofibers in Water and Wastewater Treatment. Water and Wastewater Treatment Technologies.

[16] Liao Y., Loh C.-H., Tian M., Wang R., Fane A.G. (2018). Progress in electrospun polymeric nanofibrous membranes for water treatment: Fabrication, modification and applications. Prog. Polym. Sci..

[17] Pierini F., Lanzi M., Nakielski P., Kowalewski T.A. (2017). Electrospun polyaniline-based composite nanofibers: Tuning the electrical conductivity by tailoring the structure of thiol-protected metal nanoparticles. J. Nanomater..

[18] Sagitha P., Reshmi C., Sundaran S.P., Sujith A. (2018). Recent advances in post-modification strategies of polymeric electrospun membranes. Eur. Polym. J..

[19] Yao M., Woo Y.C., Tijing L.D., Shim W.-G., Choi J.-S., Kim S.-H., Shon H.K. (2016). Effect of heat-press conditions on electrospun membranes for desalination by direct contact membrane distillation. Desalination.

[20] Kumar T.S.M., Kumar K.S., Rajini N., Siengchin S., Ayrilmis N., Rajulu A.V. (2019). A comprehensive review of electrospun nanofibers: Food and packaging perspective. Compos. Part B Eng..

[21] Wang X., Hsiao B.S. (2016). Electrospun nanofiber membranes. Curr. Opin. Chem. Eng..

[22] Thenmozhi S., Dharmaraj N., Kadirvelu K., Kim H.Y. (2017). Electrospun nanofibers: New generation materials for advanced applications. Mater. Sci. Eng. B.

[23] Esfahani H., Jose R., Ramakrishna S. (2017). Electrospun Ceramic Nanofiber Mats Today: Synthesis, Properties, and Applications. Materials (Basel).

[24] Wasim M., Sabir A., Shafiq M., Jamil T. (2019). Electrospinning: A Fiber Fabrication Technique for Water Purification. Nanoscale Materials in Water Purification.

[25] Yu M., Dong R.H., Yan X., Yu G.F., You M.H., Ning X., Long Y.Z. (2017). Recent advances in needleless electrospinning of ultrathin fibers: From academia to industrial production. Macromol. Mater. Eng..

[26] Tijing L.D., Choi W., Jiang Z., Amarjargal A., Park C.-H., Pant H.R., Im I.-T., Kim C.S. (2013). Two-nozzle electrospinning of (MWNT/PU)/PU nanofibrous composite mat with improved mechanical and thermal properties. Curr. Appl. Phys..

[27] Li Y., He J.-H. (2019). Fabrication and characterization of ZrO_2_ nanofibers by critical bubble electrospinning for high-temperature-resistant adsorption and separation. Adsorpt. Sci. Technol..

[28] Sun B., Long Y., Zhang H., Li M., Duvail J., Jiang X., Yin H. (2014). Advances in three-dimensional nanofibrous macrostructures via electrospinning. Prog. Polym. Sci..

[29] Ying Y., Ying W., Li Q., Meng D., Ren G., Yan R., Peng X. (2017). Recent advances of nanomaterial-based membrane for water purification. Appl. Mater. Today.

[30] Yang K., Dai Y., Zheng W., Ruan X., Li H., He G. (2019). ZIFs-modified GO plates for enhanced CO_2_ separation performance of ethyl cellulose based mixed matrix membranesf. Sep. Purif. Technol..

[31] Dechnik J., Gascon J., Doonan C.J., Janiak C., Sumby C.J. (2017). Mixed-matrix membranes. Angew. Chem. Int. Ed..

[32] Qadir D., Mukhtar H., Keong L.K. (2017). Mixed matrix membranes for water purification applications. Sep. Purif. Rev..

[33] Cheng Y., Ying Y., Japip S., Jiang S.-D., Chung T.-S., Zhang S., Zhao D. (2018). Advanced porous materials in mixed matrix membranes. Adv. Mater..

[34] Rezakazemi M., Sadrzadeh M., Mohammadi T., Matsuura T. (2017). Methods for the preparation of organic--inorganic nanocomposite polymer electrolyte membranes for fuel cells. Org.-Inorg. Compos. Polym. Electrolyte Membr..

[35] Chu B., Hsiao B.S., Mahajan D., Yeh T.-M. (2012). Polymeric nanofibrous composite membranes for energy efficient ethanol dehydration. J. Renew. Sustain. Energy.

[36] Vijayakumar V., Khastgir D. (2018). Hybrid composite membranes of chitosan/sulfonated polyaniline/silica as polymer electrolyte membrane for fuel cells. Carbohydr. Polym..

[37] Rezende R.A., Sabino M.A.A. (2018). From nano to macro: Enabling nanotechnologies for human organ biofabrication (Electrospun Nanofibers and Hybrid Technique). Int. J. Adv. Med Biotechnol. IJAMB.

[38] Pirzada T., Arvidson S.A., Saquing C.D., Shah S.S., Khan S.A. (2012). Hybrid silica—PVA nanofibers via sol—gel electrospinning. Langmuir.

[39] Baig M.I., Ingole P.G., Choi W.K., Jeon J.-D., Jang B., Moon J.H., Lee H.K. (2017). Synthesis and characterization of thin film nanocomposite membranes incorporated with surface functionalized Silicon nanoparticles for improved water vapor permeation performance. Chem. Eng. J..

[40] Yoo H.S., Kim T.G., Park T.G. (2009). Surface-functionalized electrospun nanofibers for tissue engineering and drug delivery. Adv. Drug Deliv. Rev..

[41] Homaeigohar S., Botcha N.K., Zarie E.S., Elbahri M. (2019). Ups and Downs of Water Photodecolorization by Nanocomposite Polymer Nanofibers. Nanomaterials (Basel).

[42] Tiraferri A., Kang Y., Giannelis E.P., Elimelech M. (2012). Superhydrophilic thin-film composite forward osmosis membranes for organic fouling control: Fouling behavior and antifouling mechanisms. Environ. Sci. Technol..

[43] Jiang S., Chen Y., Duan G., Mei C., Greiner A., Agarwal S. (2018). Electrospun nanofiber reinforced composites: A review. Polym. Chem..

[44] Pascariu P., Homocianu M. (2019). ZnO-based ceramic nanofibers: Preparation, properties and applications. Ceram. Int..

[45] Ray S.S., Chen S.-S., Nguyen N.C., Nguyen H.T. (2019). Electrospinning: A Versatile Fabrication Technique for Nanofibrous Membranes for Use in Desalination. Nanoscale Materials in Water Purification.

[46] Zahid M., Rashid A., Akram S., Rehan Z.A., Razzaq W. (2018). A comprehensive review on polymeric nano-composite membranes for water treatment. J. Membr. Sci. Technol..

[47] Methaapanon R., Chutchakul K., Pavarajarn V. (2019). Photocatalytic zinc oxide on flexible polyacrylonitrile nanofibers via sol–gel coaxial electrospinning. Ceram. Int..

[48] Xue J., Wu T., Dai Y., Xia Y. (2019). Electrospinning and Electrospun Nanofibers: Methods, Materials, and Applications. Chem. Rev..

[49] Li Z., Liu S., Song S., Xu W., Sun Y., Dai Y. (2019). Porous ceramic nanofibers as new catalysts toward heterogeneous reactions. Compos. Commun..

[50] Rodaev V.V., Razlivalova S.S., Zhigachev A.O., Vasyukov V.M., Golovin Y.I. (2019). Preparation of Zirconia Nanofibers by Electrospinning and Calcination with Zirconium Acetylacetonate as Precursor. Polymers (Basel).

[51] Xu C., Yu Z., Yuan K., Jin X., Shi S., Wang X., Zhu L., Zhang G., Xu D., Jiang H. (2019). Improved preparation of electrospun MgO ceramic fibers with mesoporous structure and the adsorption properties for lead and cadmium. Ceram. Int..

[52] Liu M., Deng N., Ju J., Fan L., Wang L., Li Z., Zhao H., Yang G., Kang W., Yan J. (2019). A Review: Electrospun Nanofiber Materials for Lithium-Sulfur Batteries. Adv. Funct. Mater..

[53] Garibay-Alvarado J., Farías R., Reyes-López S. (2019). Sol-Gel and Electrospinning Synthesis of Lithium Niobate-Silica Nanofibers. Coatings.

[54] Mercante L.A., Andre R.S., Mattoso L.H.C., Correa D.S. (2019). Electrospun Ceramic Nanofibers and Hybrid-Nanofiber Composites for Gas Sensing. ACS Appl. Nano Mater..

[55] Zahmatkesh S., Zebarjad S.M., Bahrololoom M.E., Dabiri E., Arab S.M. (2019). Synthesis of ZnO/In_2_O_3_ composite nanofibers by co-electrospinning: A comprehensive parametric investigating the process. Ceram. Int..

[56] Song X., Liu J., Wang J., Yao S., Liu B., Ma Y., Liu W., Cai Q. (2019). Non-isothermal crystallization kinetics for electrospun 3Al_2_O_3_·B_2_O_3_·2SiO_2_ ceramic nanofibers prepared using different silica sources. Ceram. Int..

[57] Yang Y., Li W., Yu D.G., Wang G., Williams G.R., Zhang Z. (2019). Tunable drug release from nanofibers coated with blank cellulose acetate layers fabricated using tri-axial electrospinning. Carbohydr. Polym..

[58] Kardani R., Asghari M., Hamedani N.F., Afsari M. (2019). Mesoporous copper zinc bimetallic imidazolate MOF as nanofiller to improve gas separation performance of PEBA-based membranes. J. Ind. Eng. Chem..

[59] Hosseinzadeh A., Zhou J.L., Altaee A., Baziar M., Li X. (2020). Modeling water flux in osmotic membrane bioreactor by adaptive network-based fuzzy inference system and artificial neural network. Bioresour. Technol..

[60] Patel S., Konar M., Sahoo H., Hota G. (2019). Surface functionalization of electrospun PAN nanofibers with ZnO-Ag heterostructure nanoparticles: Synthesis and antibacterial study. Nanotechnology.

[61] Asghari M., Sheikh M., Afsari M., Dehghani M. (2017). Molecular simulation and experimental investigation of temperature effect on chitosan-nanosilica supported mixed matrix membranes for dehydration of ethanol via pervaporation. J. Mol. Liq..

[62] Yalcinkaya F. (2019). A review on advanced nanofiber technology for membrane distillation. J. Eng. Fibers Fabr..

[63] Zhou T., Li J., Guo X., Yao Y., Zhu P., Xiang R. (2019). Freestanding PTFE electrospun tubular membrane for reverse osmosis brine concentration by vacuum membrane distillation. Desalin. Water Treat..

[64] Arribas P., García-Payo M.C., Khayet M., Gil L. (2019). Heat-treated optimized polysulfone electrospun nanofibrous membranes for high performance wastewater microfiltration. Sep. Purif. Technol..

[65] Bao T., Damtie M.M., Hosseinzadeh A., Wei W., Jin J., Vo H.N.P., Ye J.S., Liu Y., Wang X.F., Yu Z.M. (2020). Bentonite-supported nano zero-valent iron composite as a green catalyst for bisphenol A degradation: Preparation, performance, and mechanism of action. J. Environ. Manag..

[66] Liu Z., Cao R., Wei A., Zhao J., He J. (2019). Superflexible/superhydrophilic PVDF-HFP/CuO-nanosheet nanofibrous membrane for efficient microfiltration. Appl. Nanosci..

[67] Alias N.H., Jaafar J., Samitsu S., Matsuura T., Ismail A.F., Othman M.H.D., Rahman M.A., Othman N.H., Abdullah N., Paiman S.H. (2019). Photocatalytic nanofiber-coated alumina hollow fiber membranes for highly efficient oilfield produced water treatment. Chem. Eng. J..

[68] Tijing L.D., Woo Y.C., Choi J.-S., Lee S., Kim S.-H., Shon H.K. (2015). Fouling and its control in membrane distillation—A review. J. Membr. Sci..

[69] Bao T., Damtie M.M., Hosseinzadeh A., Frost R.L., Yu Z.M., Jin J., Wu K. (2020). Catalytic degradation of P-chlorophenol by muscovite-supported nano zero valent iron composite: Synthesis, characterization, and mechanism studies. Appl. Clay Sci..

[70] Tijing L.D., Yao M., Ren J., Park C.-H., Kim C.S., Shon H.K. (2019). Nanofibers for Water and Wastewater Treatment: Recent Advances and Developments. Water and Wastewater Treatment Technologies.

[71] Al-Furaiji M., Arena J.T., Ren J., Benes N., Nijmeijer A., McCutcheon J.R. (2019). Triple-layer nanofiber membranes for treating high salinity brines using direct contact membrane distillation. Membranes.

[72] Woo Y.C., Chen Y., Tijing L.D., Phuntsho S., He T., Choi J.-S., Kim S.-H., Shon H.K. (2017). CF4 plasma-modified omniphobic electrospun nanofiber membrane for produced water brine treatment by membrane distillation. J. Membr. Sci..

[73] Woo Y.C., Tijing L.D., Shim W.-G., Choi J.-S., Kim S.-H., He T., Drioli E., Shon H.K. (2016). Water desalination using graphene-enhanced electrospun nanofiber membrane via air gap membrane distillation. J. Membr. Sci..

[74] Lee D., Woo Y.C., Park K.H., Phuntsho S., Tijing L.D., Yao M., Shim W.-G., Shon H.K. (2020). Polyvinylidene fluoride phase design by two-dimensional boron nitride enables enhanced performance and stability for seawater desalination. J. Membr. Sci..

[75] Li X., Yu X., Cheng C., Deng L., Wang M., Wang X. (2015). Electrospun Superhydrophobic Organic/Inorganic Composite Nanofibrous Membranes for Membrane Distillation. ACS Appl Mater. Interfaces.

[76] Cong S., Liu X., Guo F. (2019). Membrane distillation using surface modified multi-layer porous ceramics. Int. J. Heat Mass Transf..

[77] Deka B.J., Lee E.-J., Guo J., Kharraz J., An A.K. (2019). Electrospun nanofiber membranes incorporating PDMS-aerogel superhydrophobic coating with enhanced flux and improved antiwettability in membrane distillation. Environ. Sci. Technol..

[78] Nthunya L.N., Gutierrez L., Verliefde A.R., Mhlanga S.D. (2019). Enhanced flux in direct contact membrane distillation using superhydrophobic PVDF nanofibre membranes embedded with organically modified SiO_2_ nanoparticles. J. Chem. Technol. Biotechnol..

[79] Guo J., Deka B.J., Kim K.-J., An A.K. (2019). Regeneration of superhydrophobic TiO_2_ electrospun membranes in seawater desalination by water flushing in membrane distillation. Desalination.

[80] Najafpoor A.A., Dousti S., Joneidi Jafari A., Hosseinzadeh A. (2016). Efficiency in phenol removal from aqueous solutions of pomegranate peel ash as a natural adsorbent. Environ. Health Eng. Manag. J..

[81] Tlili I., Alkanhal T.A. (2019). Nanotechnology for water purification: Electrospun nanofibrous membrane in water and wastewater treatment. J. Water Reuse Desalin..

[82] Khulbe K.C., Matsuura T. (2019). The Advances of Electrospun Nanofibers in Membrane Technology. J. Membr. Sci. Res..

[83] Zou L., Gusnawan P., Zhang G., Yu J. (2020). Novel Janus composite hollow fiber membrane-based direct contact membrane distillation (DCMD) process for produced water desalination. J. Membr. Sci..

[84] Pornea A.M., Puguan J.M.C., Deonikar V.G., Kim H. (2020). Robust Janus nanocomposite membrane with opposing surface wettability for selective oil-water separation. Sep. Purif. Technol..

[85] Nthunya L.N., Gutierrez L., Khumalo N., Derese S., Mamba B.B., Verliefde A.R., Mhlanga S.D. (2019). Superhydrophobic PVDF nanofibre membranes coated with an organic fouling resistant hydrophilic active layer for direct-contact membrane distillation. Colloids Surf. A Physicochem. Eng. Asp..

[86] Elmarghany M.R., El-Shazly A.H., Rajabzadeh S., Salem M.S., Shouman M.A., Sabry M.N., Matsuyama H., Nady N. (2020). Triple-Layer Nanocomposite Membrane Prepared by Electrospinning Based on Modified PES with Carbon Nanotubes for Membrane Distillation Applications. Membranes.

[87] Kebria M.R.S., Rahimpour A., Salestan S.K., Seyedpour S.F., Jafari A., Banisheykholeslami F., Kiadeh N.T.H. (2020). Hyper-branched dendritic structure modified PVDF electrospun membranes for air gap membrane distillation. Desalination.

[88] Wang K., Hou D., Qi P., Li K., Yuan Z., Wang J. (2019). Development of a composite membrane with underwater-oleophobic fibrous surface for robust anti-oil-fouling membrane distillation. J. Colloid Interface Sci..

[89] Pan S.-F., Dong Y., Zheng Y.-M., Zhong L.-B., Yuan Z.-H. (2017). Self-sustained hydrophilic nanofiber thin film composite forward osmosis membranes: Preparation, characterization and application for simulated antibiotic wastewater treatment. J. Membr. Sci..

[90] Shokrollahzadeh S., Tajik S. (2018). Fabrication of thin film composite forward osmosis membrane using electrospun polysulfone/polyacrylonitrile blend nanofibers as porous substrate. Desalination.

[91] Najafpoor A.A., Sadeghi A., Alidadi H., Davoudi M., Mohebrad B., Hosseinzadeh A., Jafarpour S., Zarei A. (2015). Biodegradation of high concentrations of phenol by baker’s yeast in anaerobic sequencing batch reactor. Environ. Health Eng. Manag. J..

[92] Habiba U., Afifi A.M., Salleh A., Ang B.C. (2017). Chitosan/(polyvinyl alcohol)/zeolite electrospun composite nanofibrous membrane for adsorption of Cr^6+^, Fe^3+^ and Ni^2+^. J. Hazard. Mater..

[93] Wang L., Xie Y., Liu B., Ma D., Wang X., Zhu L., Jin X., Wang Z., Xu C., Zhang G. (2019). Flexible TiO_2_ ceramic fibers near-infrared reflective membrane fabricated by electrospinning. Ceram. Int..

[94] Liu X., Jiang B., Yin X., Ma H., Hsiao B.S. (2020). Highly permeable nanofibrous composite microfiltration membranes for removal of nanoparticles and heavy metal ions. Sep. Purif. Technol..

[95] Wang C., Wang J., Zeng L., Qiao Z., Liu X., Liu H., Zhang J., Ding J. (2019). Fabrication of Electrospun Polymer Nanofibers with Diverse Morphologies. Molecules.

[96] Wang C., Sun S., Zhang L., Yin J., Jiao T., Zhang L., Xu Y., Zhou J., Peng Q. (2019). Facile preparation and catalytic performance characterization of AuNPs-loaded hierarchical electrospun composite fibers by solvent vapor annealing treatment. Colloids Surf. A Physicochem. Eng. Asp..

[97] Zhan Y., Wan X., He S., Yang Q., He Y. (2018). Design of durable and efficient poly(arylene ether nitrile)/bioinspired polydopamine coated graphene oxide nanofibrous composite membrane for anionic dyes separation. Chem. Eng. J..

[98] Karim M.R., Aijaz M.O., Alharth N.H., Alharbi H.F., Al-Mubaddel F.S., Awual M.R. (2019). Composite nanofibers membranes of poly(vinyl alcohol)/chitosan for selective lead (II) and cadmium (II) ions removal from wastewater. Ecotoxicol. Environ. Saf..

[99] Li M., Li Y., Chang K., Cheng P., Liu K., Liu Q., Wang Y., Lu Z., Wang D. (2018). The poly(vinyl alcohol-co-ethylene) nanofiber/silica coated composite membranes for oil/water and oil-in-water emulsion separation. Compos. Commun..

[100] Seyed Shahabadi S.M., Rabiee H., Seyedi S.M., Mokhtare A., Brant J.A. (2017). Superhydrophobic dual layer functionalized titanium dioxide/polyvinylidene fluoride-co-hexafluoropropylene (TiO_2_/PH) nanofibrous membrane for high flux membrane distillation. J. Membr. Sci..

[101] Chen H., Lin J., Zhang N., Chen L., Zhong S., Wang Y., Zhang W., Ling Q. (2018). Preparation of MgAl-EDTA-LDH based electrospun nanofiber membrane and its adsorption properties of copper (II) from wastewater. J. Hazard. Mater..

[102] Shalaby T., Hamad H., Ibrahim E., Mahmoud O., Al-Oufy A. (2018). Electrospun nanofibers hybrid composites membranes for highly efficient antibacterial activity. Ecotoxicol. Environ. Saf..

[103] Luo Z., Fang Q., Xu X., Raj D.V., Zhou X., Liu Z. (2019). Attapulgite nanofibers and graphene oxide composite membrane for high-performance molecular separation. J. Colloid Interface Sci..

[104] Khayet M., García-Payo M.C., García-Fernández L., Contreras-Martínez J. (2018). Dual-layered electrospun nanofibrous membranes for membrane distillation. Desalination.

[105] Lee E.-J., An A.K., Hadi P., Lee S., Woo Y.C., Shon H.K. (2017). Advanced multi-nozzle electrospun functionalized titanium dioxide/polyvinylidene fluoride-co-hexafluoropropylene (TiO_2_/PVDF-HFP) composite membranes for direct contact membrane distillation. J. Membr. Sci..

[106] Tijing L.D., Woo Y.C., Johir M.A.H., Choi J.-S., Shon H.K. (2014). A novel dual-layer bicomponent electrospun nanofibrous membrane for desalination by direct contact membrane distillation. Chem. Eng. J..

[107] Li X., García-Payo M.C., Khayet M., Wang M., Wang X. (2017). Superhydrophobic polysulfone/polydimethylsiloxane electrospun nanofibrous membranes for water desalination by direct contact membrane distillation. J. Membr. Sci..

[108] Zhu Z., Liu Y., Hou H., Shi W., Qu F., Cui F., Wang W. (2018). Dual-Bioinspired Design for Constructing Membranes with Superhydrophobicity for Direct Contact Membrane Distillation. Environ. Sci. Technol..

[109] Ray S.S., Chen S.-S., Nguyen N.C., Hsu H.-T., Nguyen H.T., Chang C.-T. (2017). Poly(vinyl alcohol) incorporated with surfactant based electrospun nanofibrous layer onto polypropylene mat for improved desalination by using membrane distillation. Desalination.

[110] Lee J.-G., Lee E.-J., Jeong S., Guo J., An A.K., Guo H., Kim J., Leiknes T., Ghaffour N. (2017). Theoretical modeling and experimental validation of transport and separation properties of carbon nanotube electrospun membrane distillation. J. Membr. Sci..

[111] Kyoungjin An A., Lee E.J., Guo J., Jeong S., Lee J.G., Ghaffour N. (2017). Enhanced vapor transport in membrane distillation via functionalized carbon nanotubes anchored into electrospun nanofibres. Sci. Rep..

[112] Tijing L.D., Woo Y.C., Shim W.-G., He T., Choi J.-S., Kim S.-H., Shon H.K. (2016). Superhydrophobic nanofiber membrane containing carbon nanotubes for high-performance direct contact membrane distillation. J. Membr. Sci..

[113] Yang F., Efome J.E., Rana D., Matsuura T., Lan C. (2018). Metal-Organic Frameworks Supported on Nanofiber for Desalination by Direct Contact Membrane Distillation. ACS Appl. Mater. Interfaces.

[114] Efome J.E., Rana D., Matsuura T., Lan C.Q. (2016). Enhanced performance of PVDF nanocomposite membrane by nanofiber coating: A membrane for sustainable desalination through MD. Water Res..

[115] Huang Y.-X., Wang Z., Hou D., Lin S. (2017). Coaxially electrospun super-amphiphobic silica-based membrane for anti-surfactant-wetting membrane distillation. J. Membr. Sci..

[116] An A.K., Guo J., Lee E.-J., Jeong S., Zhao Y., Wang Z., Leiknes T. (2017). PDMS/PVDF hybrid electrospun membrane with superhydrophobic property and drop impact dynamics for dyeing wastewater treatment using membrane distillation. J. Membr. Sci..

[117] Lee E.J., Deka B.J., Guo J., Woo Y.C., Shon H.K., An A.K. (2017). Engineering the Re-Entrant Hierarchy and Surface Energy of PDMS-PVDF Membrane for Membrane Distillation Using a Facile and Benign Microsphere Coating. Environ. Sci. Technol..

[118] Su C., Chang J., Tang K., Gao F., Li Y., Cao H. (2017). Novel three-dimensional superhydrophobic and strength-enhanced electrospun membranes for long-term membrane distillation. Sep. Purif. Technol..

[119] Lee E.-J., An A.K., He T., Woo Y.C., Shon H.K. (2016). Electrospun nanofiber membranes incorporating fluorosilane-coated TiO2 nanocomposite for direct contact membrane distillation. J. Membr. Sci..

[120] Duong H.C., Chuai D., Woo Y.C., Shon H.K., Nghiem L.D., Sencadas V. (2018). A novel electrospun, hydrophobic, and elastomeric styrene-butadiene-styrene membrane for membrane distillation applications. J. Membr. Sci..

[121] Ke H., Feldman E., Guzman P., Cole J., Wei Q., Chu B., Alkhudhiri A., Alrasheed R., Hsiao B.S. (2016). Electrospun polystyrene nanofibrous membranes for direct contact membrane distillation. J. Membr. Sci..

[122] Ren L.-F., Xia F., Chen V., Shao J., Chen R., He Y. (2017). TiO_2_-FTCS modified superhydrophobic PVDF electrospun nanofibrous membrane for desalination by direct contact membrane distillation. Desalination.

[123] Hammami M.A., Croissant J.G., Francis L., Alsaiari S.K., Anjum D.H., Ghaffour N., Khashab N.M. (2017). Engineering Hydrophobic Organosilica Nanoparticle-Doped Nanofibers for Enhanced and Fouling Resistant Membrane Distillation. ACS Appl. Mater. Interfaces.

[124] Shaulsky E., Nejati S., Boo C., Perreault F., Osuji C.O., Elimelech M. (2017). Post-fabrication modification of electrospun nanofiber mats with polymer coating for membrane distillation applications. J. Membr. Sci..

[125] An X., Liu Z., Hu Y. (2018). Amphiphobic surface modification of electrospun nanofibrous membranes for anti-wetting performance in membrane distillation. Desalination.

[126] Mohamed A., Nasser W.S., Osman T.A., Toprak M.S., Muhammed M., Uheida A. (2017). Removal of chromium (VI) from aqueous solutions using surface modified composite nanofibers. J. Colloid Interface Sci..

[127] Zhu M., Han J., Wang F., Shao W., Xiong R., Zhang Q., Pan H., Yang Y., Samal S.K., Zhang F. (2017). Electrospun nanofibers membranes for effective air filtration. Macromol. Mater. Eng..

[128] Zheng F., Zhu Z. (2018). Flexible, Freestanding, and Functional SiO_2_ Nanofibrous Mat for Dye-Sensitized Solar Cell and Photocatalytic Dye Degradation. ACS Appl. Nano Mater..

[129] Yang B.Y., Cao Y., Qi F.F., Li X.Q., Xu Q. (2015). Atrazine adsorption removal with nylon6/polypyrrole core-shell nanofibers mat: Possible mechanism and characteristics. Nanoscale Res. Lett..

[130] Mohamed A., El-Sayed R., Osman T.A., Toprak M.S., Muhammed M., Uheida A. (2016). Composite nanofibers for highly efficient photocatalytic degradation of organic dyes from contaminated water. Environ. Res..

[131] Naseri A., Samadi M., Mahmoodi N.M., Pourjavadi A., Mehdipour H., Moshfegh A.Z. (2017). Tuning Composition of Electrospun ZnO/CuO Nanofibers: Toward Controllable and Efficient Solar Photocatalytic Degradation of Organic Pollutants. J. Phys. Chem. C.

[132] Lee J., Yoon J., Kim J.-H., Lee T., Byun H. (2018). Electrospun PAN-GO composite nanofibers as water purification membranes. J. Appl. Polym. Sci..

[133] Li Z., Li T., An L., Fu P., Gao C., Zhang Z. (2016). Highly efficient chromium(VI) adsorption with nanofibrous filter paper prepared through electrospinning chitosan/polymethylmethacrylate composite. Carbohydr. Polym..

[134] Liu X., Ma H., Hsiao B.S. (2019). Interpenetrating Nanofibrous Composite Membranes for Water Purification. ACS Appl. Nano Mater..

[135] Sadeghzadeh A., Bazgir S., Shirazi M.M.A. (2020). Fabrication and characterization of a novel hydrophobic polystyrene membrane using electroblowing technique for desalination by direct contact membrane distillation. Sep. Purif. Technol..

[136] Chen Y.-P., Liu H.-Y., Liu Y.-W., Lee T.-Y., Liu S.-J. (2019). Determination of Electrospinning Parameters’ Strength in Poly(D,L)-lactide-co-glycolide Micro/Nanofiber Diameter Tailoring. J. Nanomater..

[137] Deng L., Li P., Liu K., Wang X., Hsiao B.S. (2019). Robust superhydrophobic dual layer nanofibrous composite membranes with a hierarchically structured amorphous polypropylene skin for membrane distillation. J. Mater. Chem. A.

[138] Su C., Li Y., Cao H., Lu C., Li Y., Chang J., Duan F. (2019). Novel PTFE hollow fiber membrane fabricated by emulsion electrospinning and sintering for membrane distillation. J. Membr. Sci..

[139] Jiao L., Yan K., Wang J., Lin S., Li G., Bi F., Zhang L. (2020). Low surface energy nanofibrous membrane for enhanced wetting resistance in membrane distillation process. Desalination.

[140] Li H., Shi W., Zeng X., Huang S., Zhang H., Qin X. (2020). Improved desalination properties of hydrophobic GO-incorporated PVDF electrospun nanofibrous composites for vacuum membrane distillation. Sep. Purif. Technol..

[141] Kim J., Lee J., Ha J.-H., Song I.-H. (2019). Effect of silica on flexibility of yttria-stabilized zirconia nanofibers for developing water purification membranes. Ceram. Int..

[142] Bortolassi A.C.C., Nagarajan S.A. (2019). Efficient nanoparticles removal and bactericidal action of electrospun nanofibers membranes for air filtration. Mater. Sci. Eng. C.

[143] Huang X., Jiao T., Liu Q., Zhang L., Zhou J., Li B., Peng Q. (2018). Hierarchical electrospun nanofibers treated by solvent vapor annealing as air filtration mat for high-efficiency PM2.5 capture. Sci. China Mater..

[144] Dankeaw A., Gualandris F., Silva R.H., Scipioni R., Hansen K.K., Ksapabutr B., Esposito V., Marani D. (2019). Highly porous Ce–W–TiO_2_ free-standing electrospun catalytic membranes for efficient de-NOxvia ammonia selective catalytic reduction. Environ. Sci. Nano.

[145] Wang Z., Zhao C., Pan Z. (2015). Porous bead-on-string poly(lactic acid) fibrous membranes for air filtration. J. Colloid Interface Sci..

[146] Liu C., Hsu P.C., Lee H.W., Ye M., Zheng G., Liu N., Li W., Cui Y. (2015). Transparent air filter for high-efficiency PM2.5 capture. Nat. Commun..

[147] Vitchuli N., Shi Q., Nowak J., McCord M., Bourham M., Zhang X. (2010). Electrospun ultrathin nylon fibers for protective applications. J. Appl. Polym. Sci..

[148] Sambaer W., Zatloukal M., Kimmer D., Zatloukal M. (2011). 3D Air Filtration Modeling for Nanofiber Based Filters in the Ultrafine Particle Size Range. AIP Conf. Proc..

[149] Wang N., Yang Y., Al-Deyab S.S., El-Newehy M., Yu J., Ding B. (2015). Ultra-light 3D nanofibre-nets binary structured nylon 6–polyacrylonitrile membranes for efficient filtration of fine particulate matter. J. Mater. Chem. A.

[150] Wang N., Raza A., Si Y., Yu J., Sun G., Ding B. (2013). Tortuously structured polyvinyl chloride/polyurethane fibrous membranes for high-efficiency fine particulate filtration. J. Colloid Interface Sci..

[151] Wang N., Zhu Z., Sheng J., Al-Deyab S.S., Yu J., Ding B. (2014). Superamphiphobic nanofibrous membranes for effective filtration of fine particles. J. Colloid Interface Sci..

[152] Wang N., Si Y., Wang N., Sun G., El-Newehy M., Al-Deyab S.S., Ding B. (2014). Multilevel structured polyacrylonitrile/silica nanofibrous membranes for high-performance air filtration. Sep. Purif. Technol..

[153] Chen Y., Shafiq M., Liu M., Morsi Y., Mo X. (2020). Advanced fabrication for electrospun three-dimensional nanofiber aerogels and scaffolds. Bioact. Mater..

[154] Xu T., Ding Y., Liang Z., Sun H., Zheng F., Zhu Z., Zhao Y., Fong H. (2020). Three-dimensional monolithic porous structures assembled from fragmented electrospun nanofiber mats/membranes: Methods, properties, and applications. Prog. Mater. Sci..

[155] Ren K., Wang Y., Sun T., Yue W., Zhang H. (2017). Electrospun PCL/gelatin composite nanofiber structures for effective guided bone regeneration membranes. Mater. Sci. Eng. C.

[156] Rezk A.I., Unnithan A.R., Park C.H., Kim C.S. (2018). Rational design of bone extracellular matrix mimicking tri-layered composite nanofibers for bone tissue regeneration. Chem. Eng. J..

[157] Nalvuran H., Elin A.E., Elin Y.M. (2018). Nanofibrous silk fibroin/reduced graphene oxide scaffolds for tissue engineering and cell culture applications. Int. J. Biol. Macromol..

[158] Bahrami S., Solouk A., Mirzadeh H., Seifalian A.M. (2019). Electroconductive polyurethane/graphene nanocomposite for biomedical applications. Compos. Part B Eng..

[159] Srikar R., Yarin A., Megaridis C., Bazilevsky A., Kelley E. (2008). Desorption-limited mechanism of release from polymer nanofibers. Langmuir.

[160] Garcia-Orue I., Gainza G., Garcia-Garcia P., Gutierrez F.B., Aguirre J.J., Hernandez R.M., Delgado A., Igartua M. (2019). Composite nanofibrous membranes of PLGA/Aloe vera containing lipid nanoparticles for wound dressing applications. Int. J. Pharm..

[161] Cremar L., Gutierrez J., Martinez J., Materon L., Gilkerson R., Xu F., Lozano K. (2018). Development of antimicrobial chitosan based nanofiber dressings for wound healing applications. Nanomed. J..

[162] Jaganathan S.K., Mani M.P. (2018). Electrospun polyurethane nanofibrous composite impregnated with metallic copper for wound-healing application. 3 Biotech.

[163] Kouhi M., Fathi M., Jayarama Reddy V., Ramakrishna S. (2019). Bredigite Reinforced Electrospun Nanofibers for Bone Tissue Engineering. Mater. Today Proc..

[164] Rijal N.P., Adhikari U., Khanal S., Pai D., Sankar J., Bhattarai N. (2018). Magnesium oxide-poly(ε-caprolactone)-chitosan-based composite nanofiber for tissue engineering applications. Mater. Sci. Eng. B.

[165] Jin H.-J., Fridrikh S.V., Rutledge G.C., Kaplan D.L. (2002). Electrospinning Bombyx mori silk with poly(ethylene oxide). Biomacromolecules.

[166] Lee D., Lee S.J., Moon J.-H., Kim J.H., Heo D.N., Bang J.B., Lim H.-N., Kwon I.K. (2018). Preparation of antibacterial chitosan membranes containing silver nanoparticles for dental barrier membrane applications. J. Ind. Eng. Chem..

[167] Khan M.Q., Kharaghani D., Nishat N., Shahzad A., Hussain T., Khatri Z., Zhu C., Kim I.S. (2019). Preparation and characterizations of multifunctional PVA/ZnO nanofibers composite membranes for surgical gown application. J. Mater. Res. Technol..

[168] Gu X., Li Y., Cao R., Liu S., Fu C., Feng S., Yang C., Cheng W., Wang Y. (2019). Novel electrospun poly(lactic acid)/poly(butylene carbonate)/graphene oxide nanofiber membranes for antibacterial applications. AIP Adv..

[169] Toskas G., Cherif C., Hund R.-D., Laourine E., Mahltig B., Fahmi A., Heinemann C., Hanke T. (2013). Chitosan (PEO)/silica hybrid nanofibers as a potential biomaterial for bone regeneration. Carbohydr. Polym..

[170] Waghmare V.S., Wadke P.R., Dyawanapelly S., Deshpande A., Jain R., Dandekar P. (2018). Starch based nanofibrous scaffolds for wound healing applications. Bioact. Mater..

[171] Jatoi A.W., Ogasawara H., Kim I.S., Ni Q.-Q. (2019). Polyvinyl alcohol nanofiber based three phase wound dressings for sustained wound healing applications. Mater. Lett..

[172] Sundarrajan S., Venkatesan A., Agarwal S.R., Ahamed N.N.S.A., Ramakrishna S. (2014). Fabrication of NiO/zirconium oxide nanofibers by electrospinning. Mater. Sci. Eng. C.

[173] Kida K., Chang H.-Y., Chang C.-C., Cheng L.-P. (2019). Preparation of hydrophobic nanofibers by electrospinning of PMMA dissolved in 2-propanol and water. MATEC Web Conf..

[174] Fornaguera C., Dols-Perez A., Caldero G., Garcia-Celma M.J., Camarasa J., Solans C. (2015). PLGA nanoparticles prepared by nano-emulsion templating using low-energy methods as efficient nanocarriers for drug delivery across the blood-brain barrier. J. Control. Release.

[175] Potrc T., Baumgartner S., Roskar R., Planinsek O., Lavric Z., Kristl J., Kocbek P. (2015). Electrospun polycaprolactone nanofibers as a potential oromucosal delivery system for poorly water-soluble drugs. Eur. J. Pharm. Sci..

[176] Valente T.A., Silva D.M., Gomes P.S., Fernandes M.H., Santos J.D., Sencadas V. (2016). Effect of Sterilization Methods on Electrospun Poly(lactic acid) (PLA) Fiber Alignment for Biomedical Applications. ACS Appl. Mater. Interfaces.

[177] Ghosh S.K., Adhikary P., Jana S., Biswas A., Sencadas V., Gupta S.D., Tudu B., Mandal D. (2017). Electrospun gelatin nanofiber based self-powered bio-e-skin for health care monitoring. Nano Energy.

[178] Wang W., Jin X., Zhu Y., Zhu C., Yang J., Wang H., Lin T. (2016). Effect of vapor-phase glutaraldehyde crosslinking on electrospun starch fibers. Carbohydr. Polym..

[179] Huang G.P., Shanmugasundaram S., Masih P., Pandya D., Amara S., Collins G., Arinzeh T.L. (2015). An investigation of common crosslinking agents on the stability of electrospun collagen scaffolds. J. Biomed. Mater. Res. A.

[180] Mohanty C., Sahoo S.K. (2017). Curcumin and its topical formulations for wound healing applications. Drug Discov. Today.

[181] Fukunishi T., Best C.A., Sugiura T., Shoji T., Yi T., Udelsman B., Ohst D., Ong C.S., Zhang H., Shinoka T. (2016). Tissue-Engineered Small Diameter Arterial Vascular Grafts from Cell-Free Nanofiber PCL/Chitosan Scaffolds in a Sheep Model. PLoS ONE.

[182] Baradaran-Rafii A., Biazar E., Heidari-Keshel S. (2015). Cellular Response of Limbal Stem Cells on PHBV/Gelatin Nanofibrous Scaffold for Ocular Epithelial Regeneration. Int. J. Polym. Mater. Polym. Biomater..

[183] Shao W., He J., Sang F., Ding B., Chen L., Cui S., Li K., Han Q., Tan W. (2016). Coaxial electrospun aligned tussah silk fibroin nanostructured fiber scaffolds embedded with hydroxyapatite-tussah silk fibroin nanoparticles for bone tissue engineering. Mater. Sci. Eng. C Mater. Biol. Appl..

[184] Rafienia M., Saberi A., Poorazizi E. (2017). A novel fabrication of PVA/Alginate-Bioglass electrospun for biomedical engineering application. Nanomed. J..

[185] Unnithan A.R., Sasikala A.R., Murugesan P., Gurusamy M., Wu D., Park C.H., Kim C.S. (2015). Electrospun polyurethane-dextran nanofiber mats loaded with Estradiol for post-menopausal wound dressing. Int. J. Biol. Macromol..

[186] Shao W., He J., Sang F., Wang Q., Chen L., Cui S., Ding B. (2016). Enhanced bone formation in electrospun poly(L-lactic-co-glycolic acid)-tussah silk fibroin ultrafine nanofiber scaffolds incorporated with graphene oxide. Mater. Sci. Eng. C Mater. Biol. Appl..

[187] Abdal-hay A., Abbasi N., Gwiazda M., Hamlet S., Ivanovski S. (2018). Novel polycaprolactone/hydroxyapatite nanocomposite fibrous scaffolds by direct melt-electrospinning writing. Eur. Polym. J..

[188] Apalangya V.A., Rangari V.K., Tiimob B.J., Jeelani S., Samuel T. (2019). Eggshell Based Nano-Engineered Hydroxyapatite and Poly(lactic) Acid Electrospun Fibers as Potential Tissue Scaffold. Int. J. Biomater..

[189] Kranthi Kiran A.S., Kizhakeyil A., Ramalingam R., Verma N.K., Lakshminarayanan R., Kumar T.S.S., Doble M., Ramakrishna S. (2019). Drug loaded electrospun polymer/ceramic composite nanofibrous coatings on titanium for implant related infections. Ceram. Int..

[190] Kiran A., Kumar T., Sanghavi R., Doble M., Ramakrishna S. (2018). Antibacterial and bioactive surface modifications of titanium implants by PCL/TiO_2_ nanocomposite coatings. Nanomaterials.

[191] Ye H., Zhu J., Deng D., Jin S., Li J., Man Y. (2019). Enhanced osteogenesis and angiogenesis by PCL/chitosan/Sr-doped calcium phosphate electrospun nanocomposite membrane for guided bone regeneration. J. Biomater. Sci. Polym. Ed..

[192] Toloue E.B., Karbasi S., Salehi H., Rafienia M. (2019). Potential of an electrospun composite scaffold of poly(3-hydroxybutyrate)-chitosan/alumina nanowires in bone tissue engineering applications. Mater. Sci. Eng. C Mater. Biol. Appl..

[193] Guo A., Roso M., Colombo P., Liu J., Modesti M. (2015). In situ carbon thermal reduction method for the production of electrospun metal/SiOC composite fibers. J. Mater. Sci..

[194] Wang H., Li X., Zhuang X., Cheng B., Wang W., Kang W., Shi L., Li H. (2017). Modification of Nafion membrane with biofunctional SiO_2_ nanofiber for proton exchange membrane fuel cells. J. Power Sources.

[195] Kong L., Yan Y., Qiu Z., Zhou Z., Hu J. (2018). Robust fluorinated polyimide nanofibers membrane for high-performance lithium-ion batteries. J. Membr. Sci..

[196] Wang F., Wu Y., Huang Y., Liu L. (2018). Strong, transparent and flexible aramid nanofiber/POSS hybrid organic/inorganic nanocomposite membranes. Compos. Sci. Technol..

[197] Wang H., Zhang S., Zhu M., Sui G., Yang X. (2018). Remarkable heat-resistant halloysite nanotube/polyetherimide composite nanofiber membranes for high performance gel polymer electrolyte in lithium ion batteries. J. Electroanal. Chem..

[198] Lu X., Wang C., Favier F., Pinna N. (2017). Electrospun nanomaterials for supercapacitor electrodes: Designed architectures and electrochemical performance. Adv. Energy Mater..

[199] Chinnappan A., Baskar C., Baskar S., Ratheesh G., Ramakrishna S. (2017). An overview of electrospun nanofibers and their application in energy storage, sensors and wearable/flexible electronics. J. Mater. Chem. C.

[200] Bairagi S., Ali S.W. (2019). A unique piezoelectric nanogenerator composed of melt-spun PVDF/KNN nanorod-based nanocomposite fibre. Eur. Polym. J..

[201] Guan Y., Zhang L., Li M., West J.L., Fu S. (2018). Preparation of temperature-response fibers with cholesteric liquid crystal dispersion. Colloids Surf. A Physicochem. Eng. Asp..

[202] Andre R.S., Mercante L.A., Facure M.H.M., Mattoso L.H.C., Correa D.S. (2019). Enhanced and selective ammonia detection using In2O3/reduced graphene oxide hybrid nanofibers. Appl. Surf. Sci..

[203] Khalil A., Kim J.J., Tuller H.L., Rutledge G.C., Hashaikeh R. (2016). Gas sensing behavior of electrospun nickel oxide nanofibers: Effect of morphology and microstructure. Sens. Actuators B Chem..

[204] Ab Kadir R., Li Z., Sadek A.Z., Abdul Rani R., Zoolfakar A.S., Field M.R., Ou J.Z., Chrimes A.F., Kalantar-zadeh K. (2014). Electrospun granular hollow SnO_2_ nanofibers hydrogen gas sensors operating at low temperatures. J. Phys. Chem. C.

[205] Ding Y., Hou H., Zhao Y., Zhu Z., Fong H. (2016). Electrospun polyimide nanofibers and their applications. Prog. Polym. Sci..

